# Transient receptor potential vanilloid 4 (TRPV4) channels mediate pulmonary surfactant protein A and D secretion

**DOI:** 10.1093/ajrcmb/aanag023

**Published:** 2026-02-21

**Authors:** Philipp Alt, Isabel Müller, Martina Kiefmann, Thomas Gudermann, Wolfgang M Kuebler, Matthias Griese, Claudia A Staab-Weijnitz, Alexander Dietrich

**Affiliations:** Walther Straub Institute of Pharmacology and Toxicology, Medical Faculty, LMU-Munich, Munich Germany; Walther Straub Institute of Pharmacology and Toxicology, Medical Faculty, LMU-Munich, Munich Germany; Comprehensive Pneumology Center Munich (CPC-M), Deutsches Zentrum für Lungenforschung (DZL), Munich, Germany; Walther Straub Institute of Pharmacology and Toxicology, Medical Faculty, LMU-Munich, Munich Germany; Comprehensive Pneumology Center Munich (CPC-M), Deutsches Zentrum für Lungenforschung (DZL), Munich, Germany; Walther Straub Institute of Pharmacology and Toxicology, Medical Faculty, LMU-Munich, Munich Germany; Comprehensive Pneumology Center Munich (CPC-M), Deutsches Zentrum für Lungenforschung (DZL), Munich, Germany; Institute of Physiology, Charité–Universitätsmedizin Berlin, Berlin, Germany; Comprehensive Pneumology Center Munich (CPC-M), Deutsches Zentrum für Lungenforschung (DZL), Munich, Germany; Department of Pediatrics, Dr. von Hauner Children’s Hospital, University Hospital, LMU-Munich, Munich, Germany; Comprehensive Pneumology Center Munich (CPC-M), Deutsches Zentrum für Lungenforschung (DZL), Munich, Germany; Department of Pediatrics and Division of Pulmonary Allergy and Critical Care Medicine, School of Medicine, University of Colorado Anschutz Medical Campus, Aurora, CO, United States; Walther Straub Institute of Pharmacology and Toxicology, Medical Faculty, LMU-Munich, Munich Germany; Comprehensive Pneumology Center Munich (CPC-M), Deutsches Zentrum für Lungenforschung (DZL), Munich, Germany

**Keywords:** AT2 cells, club cells, surfactant protein secretion, human and mouse air-liquid interface models

## Abstract

Lung surfactant not only reduces surface tension at the air-liquid interface (ALI), but is also involved in pulmonary host defense. This important role in innate immunity of the respiratory tract is primarily mediated by surfactant proteins A and D (SP-A, SP-D), which are secreted from alveolar epithelial type 2 (AT2) cells and from tracheal and bronchial epithelial cells expressing transient receptor potential vanilloid 4 (TRPV4) channels. In a mouse model deficient in TRPV4 (TRPV4^-/-^), reduced levels of SP-A and SP-D were detected in the bronchoalveolar lavage fluid. Production of both proteins in TRPV4^-/-^ AT2 cells was not different to wild-type control cells, but secretion of SP-A and -D was impaired both in TRPV4-deficient murine AT2 and murine tracheal epithelial cells cultured at the ALI. In a translational approach, we established a human ALI model and differentiated bronchial basal cells to a pseudostratified epithelium. Downregulation of *Trpv4* mRNA expression by specific siRNAs also resulted in a reduction of secreted SP-A levels. Interestingly, differentiation of basal cells to ciliated cells, but not club cells, which secrete SP-A and SP-D, was decreased after downregulation of TRPV4. Our data highlight novel essential functions of TRPV4 channels in secretion of SP-A and SP-D, which are important not only for innate immunity, but also for lung diseases like asthma and idiopathic pulmonary fibrosis.

Clinical Relevance Our data provide evidence for an essential role of transient receptor potential vanilloid 4 (TRPV4) channels in secretion of surfactant proteins A and D (SP-A, SP-D) in the alveolus and the murine and human tracheobronchial tree. TRPV4 protein might enhance SP-A and SP-D secretion for a more powerful immune response.

## Introduction

Our respiratory tract is responsible for gas exchange but also must be protected from invading pathogens. To facilitate both, distal pulmonary tissues produce a fluid called lung surfactant. This surfactant is mainly composed of lipids, but also contains a total of 8% of surfactant proteins A through D (SP-A, -B, -C, -D).^1^ While SP-B and -C are hydrophobic and strongly interact with the phospholipid bilayer of the surfactant,^2^ which is essential for surface tension reduction and efficient gas exchange in the alveoli of the lungs, the more hydrophilic SP-A and -D bind to apoptotic cells^3^^,^^4^ and a variety of bacteria as well as viruses, including SARS-CoV-2.^5^ Coupling to these pathogens will result in lysis, increased phagocytosis, and production of cytokines as well as reactive species by immune cells.^4^^,^^6^^,^^7^ Along this line, increased bronchoalveolar levels of SP-D have been demonstrated in children with asthma or recurrent bronchitis, while patients with an SP-D deficiency suffered from pneumonia.^8^ Recently, roles for SP-A in asthma,^9^ lung repair by macrophages,^10^ and synergistic actions with antibiotics against gram-negative respiratory bacteria^11^ were reported, while SP-D is involved in tumor immunoregulation.^12^ Both surfactant proteins (SPs) contribute to phagocytosis of nanomaterials by macrophages^13^ and are associated with infections by respiratory syncytial virus (RSV) in children,^14^ idiopathic pulmonary fibrosis (IPF), and hypersensitivity pneumonitis.^15^ All 4 SPs are produced in alveolar epithelial type 2 (AT2) cells, but SP-A and -D are also synthesized and secreted in the tracheal-bronchial region by club and submucosal cells^16^ as well as in extrapulmonary tissues.^3^ The tracheal and bronchial regions of the airways enable the removal of pathogens and debris by mucociliary clearance (previously reviewed^17^). While goblet cells mainly produce mucins, which form mucus for the mucociliary movement by ciliated cells, club cells are also able to secrete SP-A and -D for an efficient removal of invading pathogens.^18^ Along these lines, mouse models deficient in SP-A and SP-D expose a defective or altered response after a challenge with bacterial, fungal, or viral microorganisms or to bacterial lipopolysaccharides in vivo (previously reviewed^18^).

Transient receptor potential (TRP) channels, originally cloned in *Drosophila melanogaster*, serve multiple functions in different mammalian tissues,^19^ including the lung.^20^ Transient receptor potential vanilloid 4 (TRPV4) is the fourth member of the vanilloid family of TRP channels.^21^ Like most TRP channels, TRPV4 harbors an invariant sequence, the TRP box (containing the amino acid sequence EWKFAR), in its intracellular C-terminal tail as well as ankyrin repeats in the intracellular N-terminus. The protein is composed of 6 membrane-spanning helices (S1-S6) and a presumed pore-forming loop between S5 and S6.^21^^,^^22^ Four of these monomers of the same type preferentially assemble in a functional homotetrameric complex,^23^ although in cell cilia of renal epithelial cells TRPV4/TRPP2 complexes were also identified.^24^ Homotetrameric TRPV4 was originally characterized as a sensor of extracellular osmolarity.^25^^,^^26^ The channel is functionally expressed in endothelial^27^^,^^28^ and epithelial cells of the respiratory system.^29-31^ TRPV4 channels may serve as mechanosensors because they are activated by membrane and shear stretch as well as by viscous loading.^32^ Along this line, TRPV4 channels regulate ciliary beat function for mucociliary clearance by ciliated cells^29^ and are involved in pulmonary hypertension.^33^^,^^34^ Moreover, TRPV4^-/-^ mice were protected from bleomycin-induced pulmonary fibrosis due to the channel’s constitutive expression and function in lung fibroblasts.^35^ Therefore, the channel is an interesting pharmacological target and numerous modulators have already been identified in a review.^36^

In this study, we identified reduced SP-A and SP-D levels in bronchoalveolar lavage fluid (BALF) from TRPV4^-/-^ mice, which was not due to a decreased production in AT2 cells, but to a reduced secretion from AT2 cells and from differentiated mouse tracheal epithelial cells (mTECs). In a translational approach, we utilized a human air-liquid interface (ALI) model to reproduce reduced SP-A and -D expression in the bronchial region after downregulation of TRPV4 channels. Moreover, differentiation of ciliated cells, but not goblet and club cells, was reduced after TRPV4 downregulation. Therefore, our data suggest essential roles of TRPV4 channels in AT2 cells and the bronchial epithelium for the secretion of SP-A and -D.

## Materials and methods

### Animals

TRPV4^-/-^ mice (B6.199X1-Trpv4^tm1MSZ^ from Riken BioResource Center, RBRC01939)^37^^,^^38^ were backcrossed 10 times to the C57/BL6J strain. Male and female mice 3 months of age were used in the experiments, if not mentioned otherwise in the figure legends. All animal experiments were approved by the local authority (Regierung Oberbayern).

### Human ALI model

Human bronchial epithelial cells (HBECs) from healthy donors were obtained from Lonza (Basel, Switzerland, #CC2540S; see Table S1 for donor information). Ethics statements were provided by the supplier. Cells were expanded for a maximum of 3 passages in PneumaCult Ex Plus medium and were seeded onto 12-well Transwell inserts (100 000 cells/insert) with collagen IV (Sigma-Aldrich, Germany, #C6745) coating. They were kept at 37 °C and 5% CO_2_ and air lift was performed 2-3 days after seeding when confluency was reached. The medium at the basal compartment was changed to PneumaCult ALI medium and, from then, medium change was performed every 2 days. Cells were washed once a week to remove secreted mucus and wash-offs were collected for further analysis. Depending on the experimental setup, cells were cultured for 7, 14, 21, 28, or 35 days.

#### Transient knockdown of TRPV4 in HBECs

The transfection of HBECs with specific siRNAs (Horizon Discovery, United Kingdom, TRPV4 smartpool, #L-004195-00-005) against TRPV4 was performed using siRNA (Tables S2 and S3) at a final concentration of 10 nM in a mix of OptiMEM (Gibco, Thermo Fisher Scientific, USA, #31985070) and Lipofectamine RNAiMAX (Thermo Fisher Scientific, #13778030), which was applied to the apical side of the inserts with seeded cells in submerged culture with PneumaCult ExPlus medium. After transfection time of 8 hours, medium was changed on both sides of the insert and cells were incubated at 37 °C with PneumaCult ExPlus medium until air lift was performed. Successful transfection was validated via Western blot with samples taken 3, 5, and 10 days after treatment. Cells were cultured and differentiated as described above.

### Proximity ligation assay

To confirm findings of the co-immunoprecipitation experiments in the surfactant-producing cells, AT2-cells were isolated from mice and fixed 24 hours after isolation with 4% paraformaldehyde solution. The cells were treated according to the steps of the manufacturer’s protocol. In brief, cells were blocked in blocking solution and incubated with primary antibodies (from different hosts) against SP-A and TRPV4 or SP-D and TRPV4. Then, proximity ligation assay (PLA) probes and ligation buffer for rolling circle amplification were added. After amplification step and incubation with fluorescent-labeled oligonucleotide probes (complementary to the amplified product), cells were washed and mounted in DAPI-containing mounting medium (Merck, Germany, anti-mouse MINUS #DUO92004-30RXN, anti-rabbit PLUS #DUO92002-30RXN, anti-goat PLUS #DUO92003-30RXN, Duolink In Situ Mounting Medium #DUO82040-5ML; see also Table S5). For experiments shown in Figure S7, plasma membranes of cells were additionally stained with a fluorescent-coupled E-cadherin (anti-CD324; Thermo Fisher Scientific, USA, #53-3249-82) antibody. After solidifying overnight, the cells were imaged with the respective channel with a confocal microscope (LSM 880, Zeiss, Germany).

### Statistical analysis

For statistical analysis, GraphPad Prism 10 software (GraphPad Software, USA) was used. Significant differences are indicated by asterisks. All data are represented as mean + SEM.

All other methods are described in the Supplementary Materials.

## Results

### Ablation of TRPV4 decreases amounts of SP-A and SP-D in murine lung lysates and the BALF, due to decreased secretion but not to changes in their synthesis in AT2 cells

**Figure 1:**
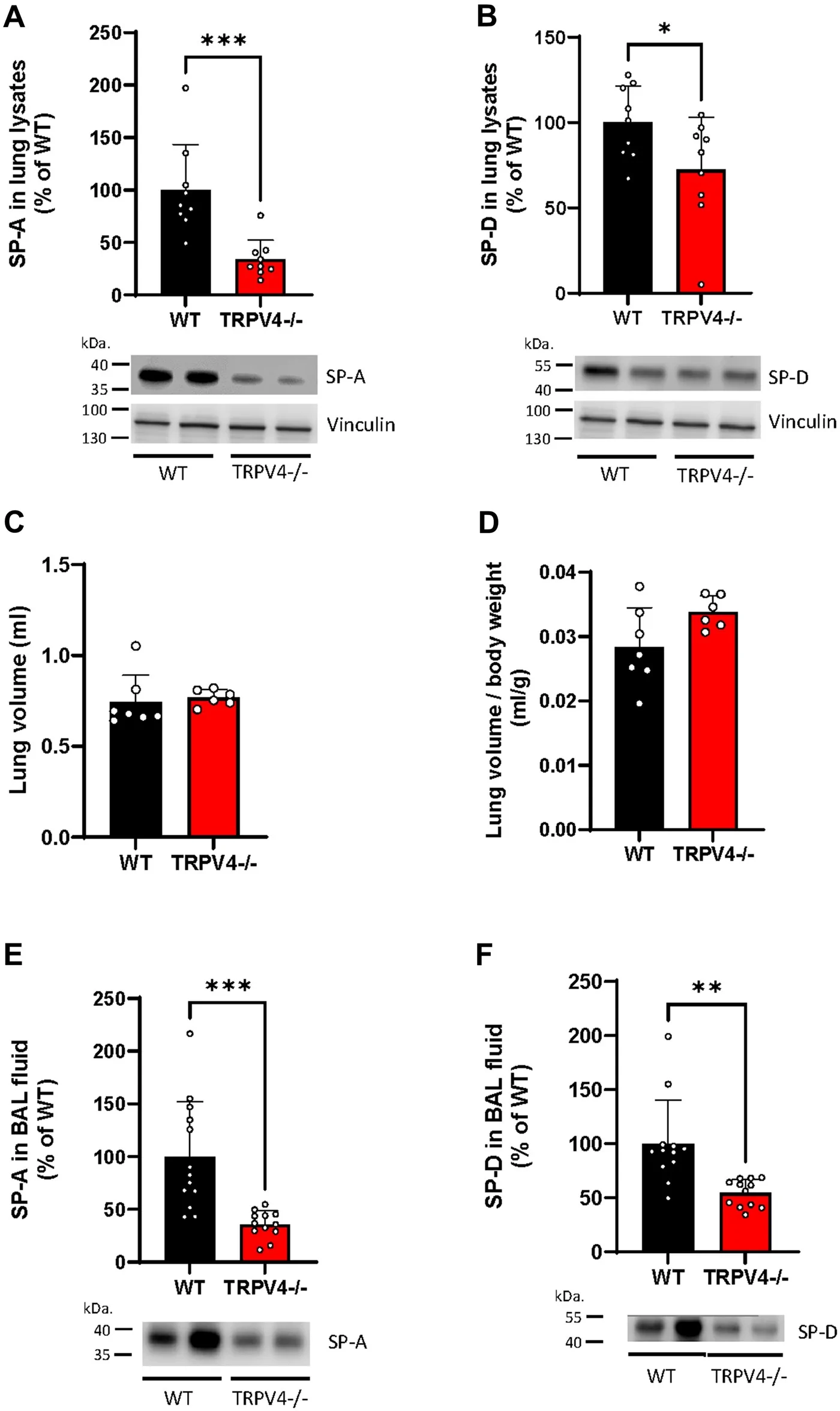
Surfactant protein A (SP-A) and D (SP-D) levels in murine lung lysates and bronchoalveolar lavage (BAL) fluid of wild-type (WT) and transient receptor potential vanilloid 4–deficient (TRPV4^–/–^) mice. (A) SP-A and (B) SP-D expression in whole mouse lungs was evaluated by immunoblotting of whole lung lysates from WT and TRPV4^–/–^ mice. Values were normalized to vinculin as loading control and are shown as % of WT. (C) Total lung volumes and (D) lung volumes normalized to body weights (lung volumes/body weight) of WT and TRPV4^–/–^ mice. (E) SP-A and (F) SP-D expression in BAL fluid from mouse lungs of WT and TRPV4^–/–^ mice. Values were normalized to lung volumes/body weight. Data represent means + SEM from at least 6-12 mice each genotype. Significance between means was analyzed using 2-tailed unpaired Student t-test: *P <.05, **P <.01, ***P <.005.

**Figure 2:**
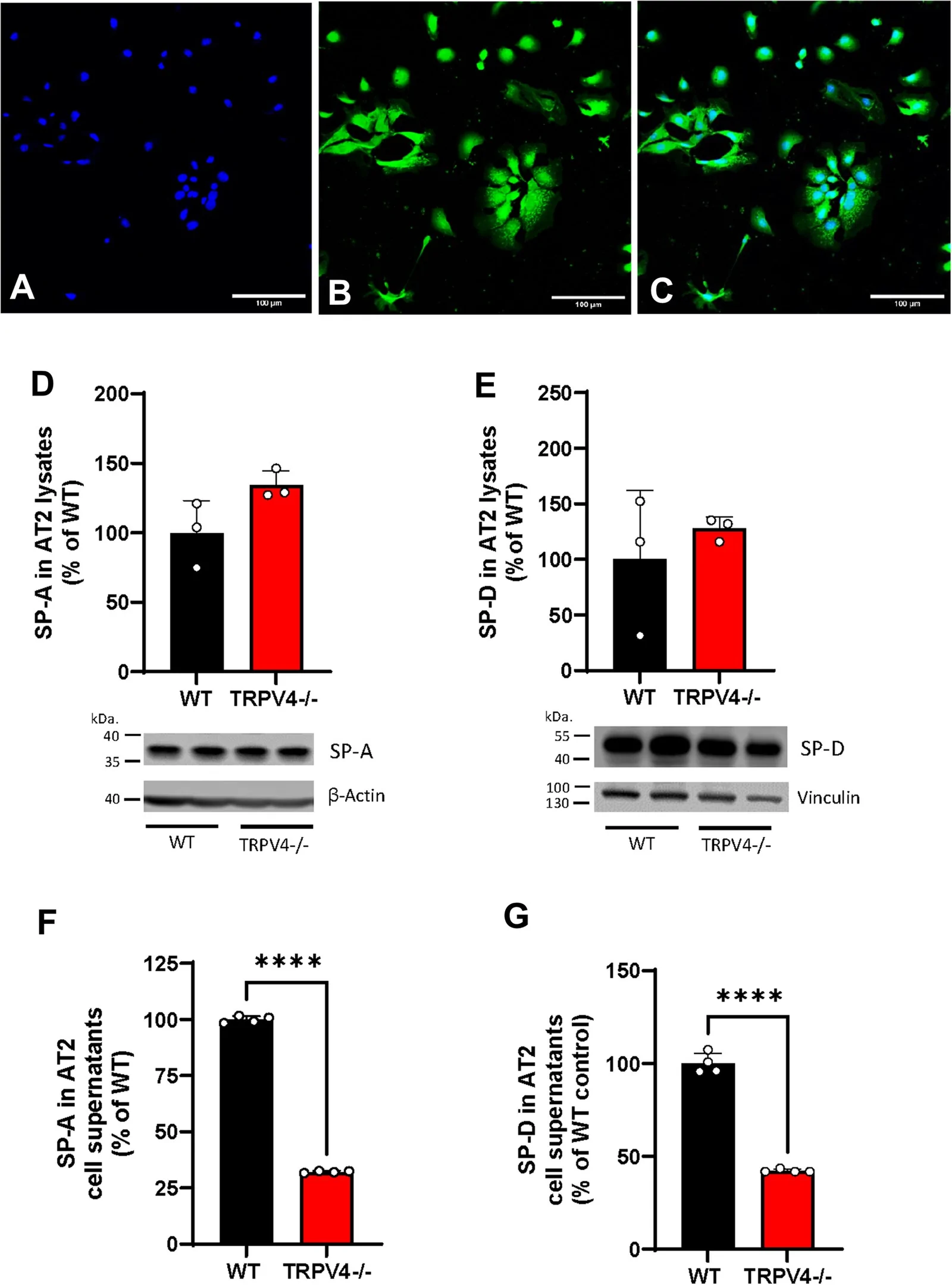
Surfactant protein A (SP-A) and D (SP-D) expression in murine alveolar type 2 (AT2) cells from wild-type (WT) and transient receptor potential vanilloid 4–deficient (TRPV4^–/–^) mice. (A and B) AT2 cells after isolation from WT mice are stained with DAPI (blue) (A) or with a pro-surfactant protein C (pSP-C) antibody with secondary anti-rabbit IgG Alexa 488 (green) (B). (C) Overlay of the 2 immunofluorescent images. (D) SP-A and (E) SP-D expression in AT2 cells was evaluated by immunoblotting of cell lysates from WT and TRPV4^–/–^ mice. Values were normalized to β-actin or vinculin as loading control and are shown as % of WT. (F) SP-A and (G) SP-D levels in AT2 cell supernatants from WT and TRPV4^–/–^ mice were quantified by ELISA. Data represent mean + SEM from 3 different isolations containing 5 mice each (total WT, *n* = 15; TRPV4^–/–^, *n* = 15). Significance between means was analyzed using 2-tailed unpaired Student t-test: ****P <.001.

To investigate the role of TRPV4 in the production and secretion of SP-A and SP-D, we isolated lungs from wild-type (WT) and TRPV4-deficient (TRPV4^-/-^) mice. A quantitative Western blot analysis of whole lung lysates revealed significantly reduced levels for both SPs in TRPV4^-/-^ mice compared to WT controls (Figure 1A, B). We next analyzed the content of both proteins in BALFs. As volumes of BALF correlate with lung volumes, we performed in vivo lung function measurements of WT and TRPV4^-/-^ mice and were able to exclude any changes in total lung volumes as well as lung volumes normalized to body weights (Figure 1C, D). SP-A and SP-D levels were significantly decreased in BALF of TRPV4^-/-^ mice compared with WT control animals (Figure 1E, F). While sex-specific differences for both SP levels were not observed in WT and TRPV4^-/-^ mice (Figure S1), only SP-A and -D levels in lung lysates of females were not significantly different in TRPV4^-/-^ compared to WT lungs (Figure S2) for yet unknown reasons. To further investigate the role of TRPV4 channels for the production of SPs, we isolated surfactant-producing AT2 cells as described previously.^39^ After identification of primary AT2 cells by its primary marker protein pro-SP-C (Figure 2A-C), we performed Ca^2+^ imaging experiments with fully differentiated AT2 cells identified by staining lamellar bodies with LysoTracker DND 26 (Figure S3A) using a TRPV4 activator (GSK 1016790A, Figure S3B) and preincubation with a TRPV4 inhibitor (HC0974047, Figure S3C) to verify again functional expression of TRPV4 channels in these cells. TRPV4 protein was not detectable in AT2 cells isolated from TRPV4^-/-^ mice (Figure S4), which showed no gross morphological changes compared to WT cells. However, expression of both SP-A and SP-D was not decreased in AT2 cells from TRPV4^-/-^ mice compared to WT mice (Figure 2D, E). Therefore, we quantified SP-A and SP-D levels in the supernatant of primary AT2 cells by ELISA and detected significant less protein secreted by TRPV4-deficient cells compared to WT controls (Figure 2F, G).

### TRPV4 channels interact with SP-A and SP-D in primary murine AT2 cells

**Figure 3:**
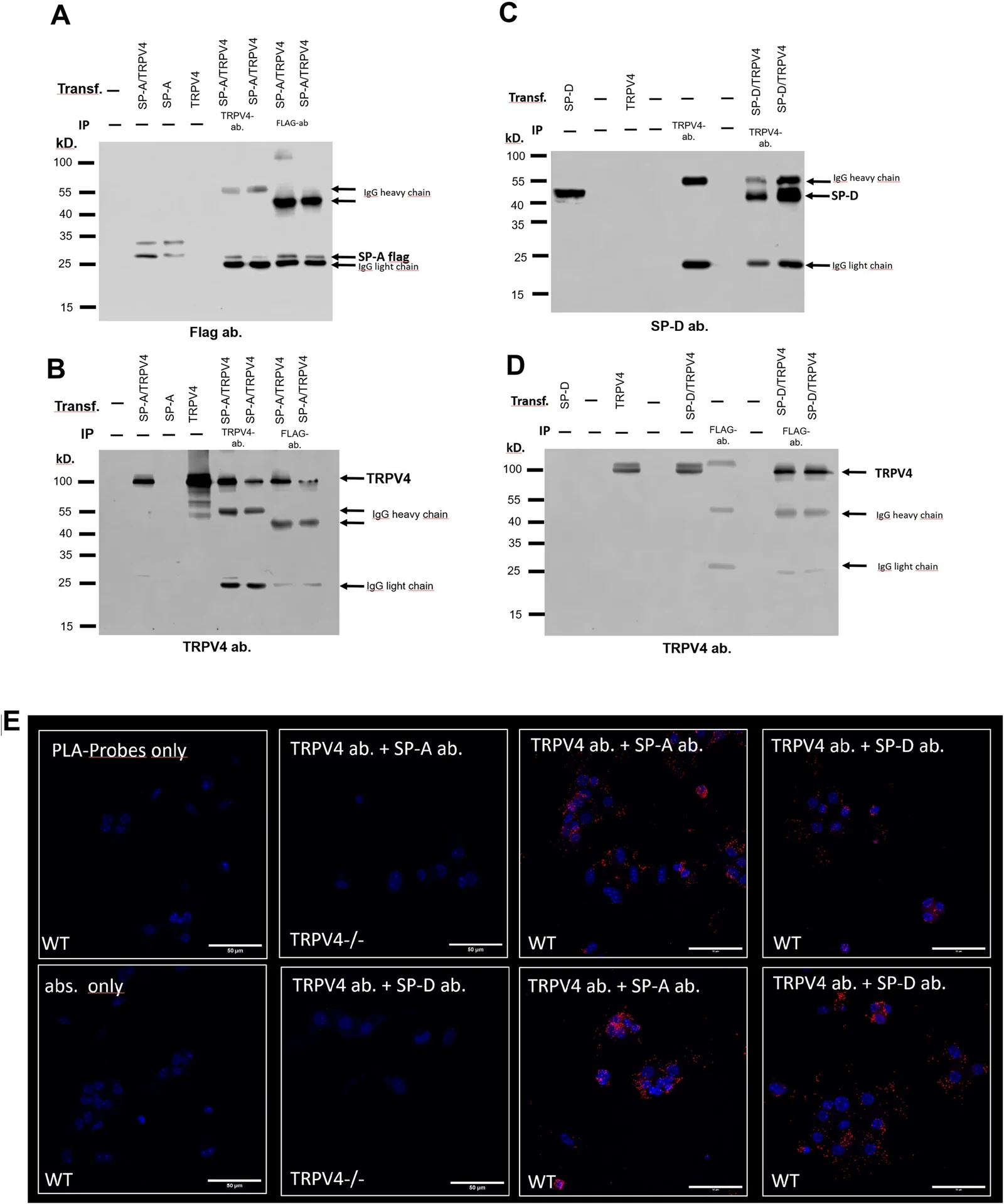
Surfactant protein A (SP-A) and D (SP-D) physically interact with transient receptor potential vanilloid 4 (TRPV4) channels in a heterologous overexpression system and primary alveolar type 2 (AT2) cells. (A) HEK293 cells were transfected with SP-A flag (SP-A), TRPV4 (TRPV4), both (SP-A/TRPV4) cDNAs, or mock (–). Proteins were co-immunoprecipitated with TRPV4 or flag antibody, separated by polyacrylamide (PAA) gel electrophoresis, and identified in a Western blot by flag antibodies (Flag ab.). (B) HEK293 cells were transfected with SP-A flag (SP-A), TRPV4 (TRPV4), both (SP-A/TRPV4) cDNAs, or mock (–). Proteins were co-immunoprecipitated with TRPV4 or flag antibody, separated by PAA gel electrophoresis, and identified in a Western blot by TRPV4 antibodies (TRPV4 ab.). (C) HEK293 cells were transfected with SP-D (SP-D), TRPV4 (TRPV4), both (SP-D/TRPV4) cDNAs, or mock (–). Proteins were co-immunoprecipitated with TRPV4 or flag antibody, separated by PAA gel electrophoresis, and identified in a Western blot by SP-D antibodies (SP-D ab.). (D) HEK293 cells were transfected with SP-D (SP-D), TRPV4 (TRPV4), both (SP-D/TRPV4) cDNAs, or mock (–). Proteins were co-immunoprecipitated with TRPV4 or flag antibody, separated by PAA gel electrophoresis, and identified in a Western blot by TRPV4 antibodies (TRPV4 ab.). (E) Fluorescent images from primary AT2 cells from wild-type (WT) and TRPV4-deficient mice (TRPV4^–/–^) after performing a proximity ligation assay (PLA) using TRPV4 (TRPV4 ab.), SP-A antibodies (SP-A ab.), SP-D antibodies (SP-D ab.), or PLA probes or antibodies only (abs only).

As both proteins are secreted independently from lamellar bodies^40^^,^^41^ in clear contrast to SP-B and SP-C, we next tested a possible interaction of TRPV4 channels with SP-A and SP-D, which might facilitate secretion of both proteins from AT2 cells. We first utilized a heterologous overexpression system in HEK293 cells and co-immunoprecipitated TRPV4 channels with flag tagged SP-A and SP-D and vice versa using a specific TRPV4 antiserum and a flag antibody, respectively (Figure 3A-D). While both proteins were co-immunoprecipitated with a TRPV4 antiserum as well as TRPV4 channels by antibodies directed against the flag tag of both SPs, another channel, TRPC6, was not able to interact with SP-A and SP-D (Figure S5). To confirm these results in primary AT2 cells, we used a proximity ligation assay (PLA), which allows detection of single protein–protein interaction by a fluorescence signal in primary cells^42^ with 2 specific antibodies detecting SP-A and SP-D in AT2 cells (Figure S6). PLA technology uses a pair of secondary antibodies tagged with oligonucleotides, which if localized within 40 nm in a cell, initiate a rolling circle amplification to be detected by fluorescence-coupled probes. Indeed, both SP-A and SP-D colocalized with TRPV4 channels in primary AT2 cells detected by fluorescence signals in WT but not in TRPV4-deficient cells (Figure 3E). To further localize this interaction of SP-A or SP-D with TRPV4 channels in AT2 cells, we used an E-cadherin antibody as a marker of cell plasma membranes after performing a PLA. Most interestingly, almost all PLA signals were localized intracellular and not at the plasma membrane (Figure S7). Therefore, TRPV4 channels might facilitate SP-A and SP-D secretion by physically interacting mostly in intracellular compartments.

### TRPV4 deficiency decreases amounts of SP-A, and SP-D in mucous layers of mTECs differentiated in an ALI model

**Figure 4:**
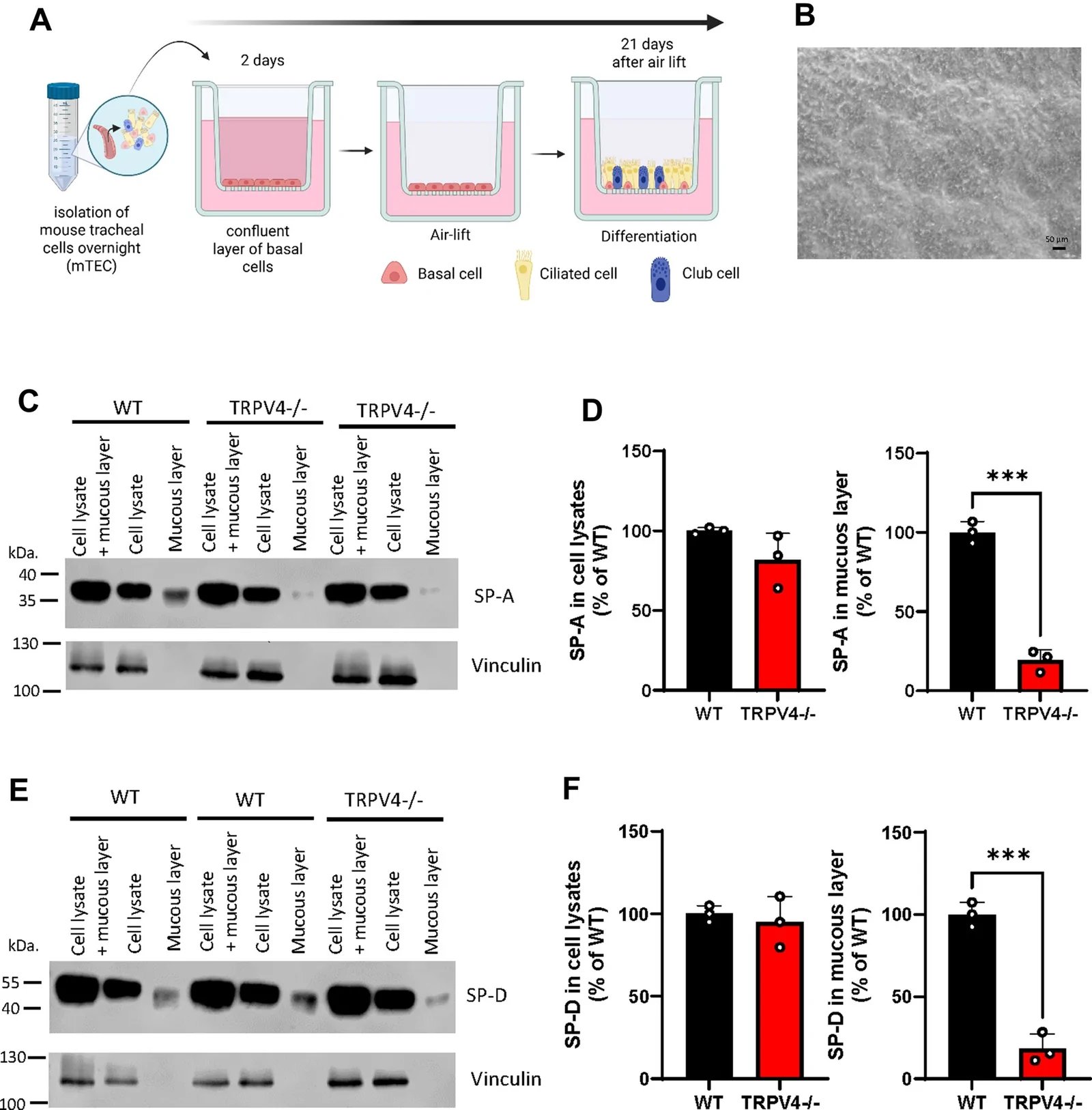
Surfactant protein A (SP-A) and D (SP-D) levels in cell lysates and mucous layers of an air-liquid interface (ALI) model with murine tracheal epithelial cells (mTECs) from wild-type (WT) and transient receptor potential vanilloid 4–deficient (TRPV4^–/–^) mice. (A) Schematic presentation of the ALI model for differentiation of club and ciliated cells from mTECs. (B) Top view on the differentiated pseudostratified epithelium with a mucous layer 21 days after air lift. (C) SP-A expression in cell lysates, cell lysates with the mucous layer, and the mucous layer of WT and TRPV4^–/–^ mTECs differentiated 21 days after air lift. Vinculin serves as loading controls for cell lysates. (D) Quantification of SP-A production in cell lysates and mucous layers of WT and TRPV4^–/–^ mTECs differentiated 21 days after air lift. (E) SP-D expression in cell lysates, cell lysates with mucous layer, and mucous layer of WT and TRPV4^–/–^ mTECs differentiated 21 days after air lift. Vinculin served as loading controls for cell lysates. (F) Quantification of SP-D production in cell lysates and mucous layer of WT and TRPV4^–/–^ mTECs differentiated 21 days after air lift. Data represent mean + SEM from at least 3 cell isolations of each genotype. Significance between means was analyzed using 2-tailed unpaired Student t-test: ***P <.001.

Next to AT2 cells, SPs, especially SP-A and -D, are also produced and secreted in club cells of the human tracheal and bronchial regions.^43^^,^^44^ To quantify SP production in these cells, we first isolated mTECs from WT and TRPV4^-/-^ mice and seeded them on cell culture inserts for expansion and differentiation in an ALI model to ciliated and club cells as described previously^45^ (Figure 4A, B). We detected no gross morphological changes in the differentiation of TRPV4^-/-^ mTECs compared to WT cells. Again, quantification of SP-A and SP-D expression in cell lysates from ALI-differentiated cells revealed no differences (Figure 4C-F). However, in mucous layers secreted from TRPV4^-/-^ cells analyzed by Western blotting, we identified significantly lower SP-A and SP-D levels compared to WT controls (Figure 4C-F). Therefore, secretion but not production of SP-A and SP-D is reduced by ablation of TRPV4 channels in the tracheal region.

### Downregulation of TRPV4 channels inhibits secretion of SP-A and basal cell differentiation to ciliated cells in an ALI model of HBECs

**Figure 5:**
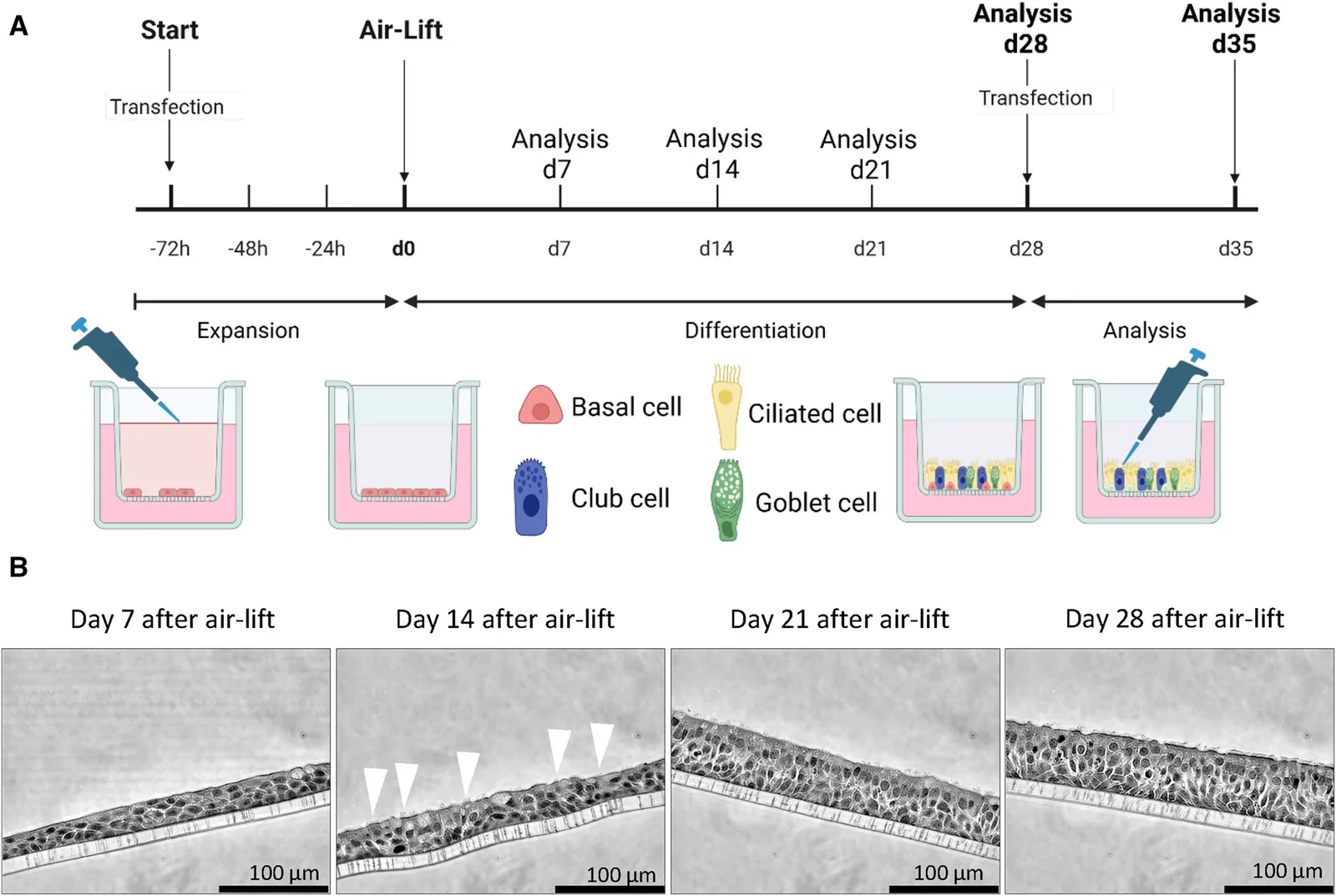
Differentiation of respiratory epithelial cells in an air-liquid interface (ALI) model with human bronchial epithelial cells (HBECs). (A) Schematic presentation of the ALI model from HBEC. Time points for transfection and analysis are marked. (B) Images from cross-sections through Transwell inserts with attached cell layers 7, 14, 21, and 28 days after air lift. Appearance of cilia from day 14 on are marked by white arrowheads.

**Figure 6:**
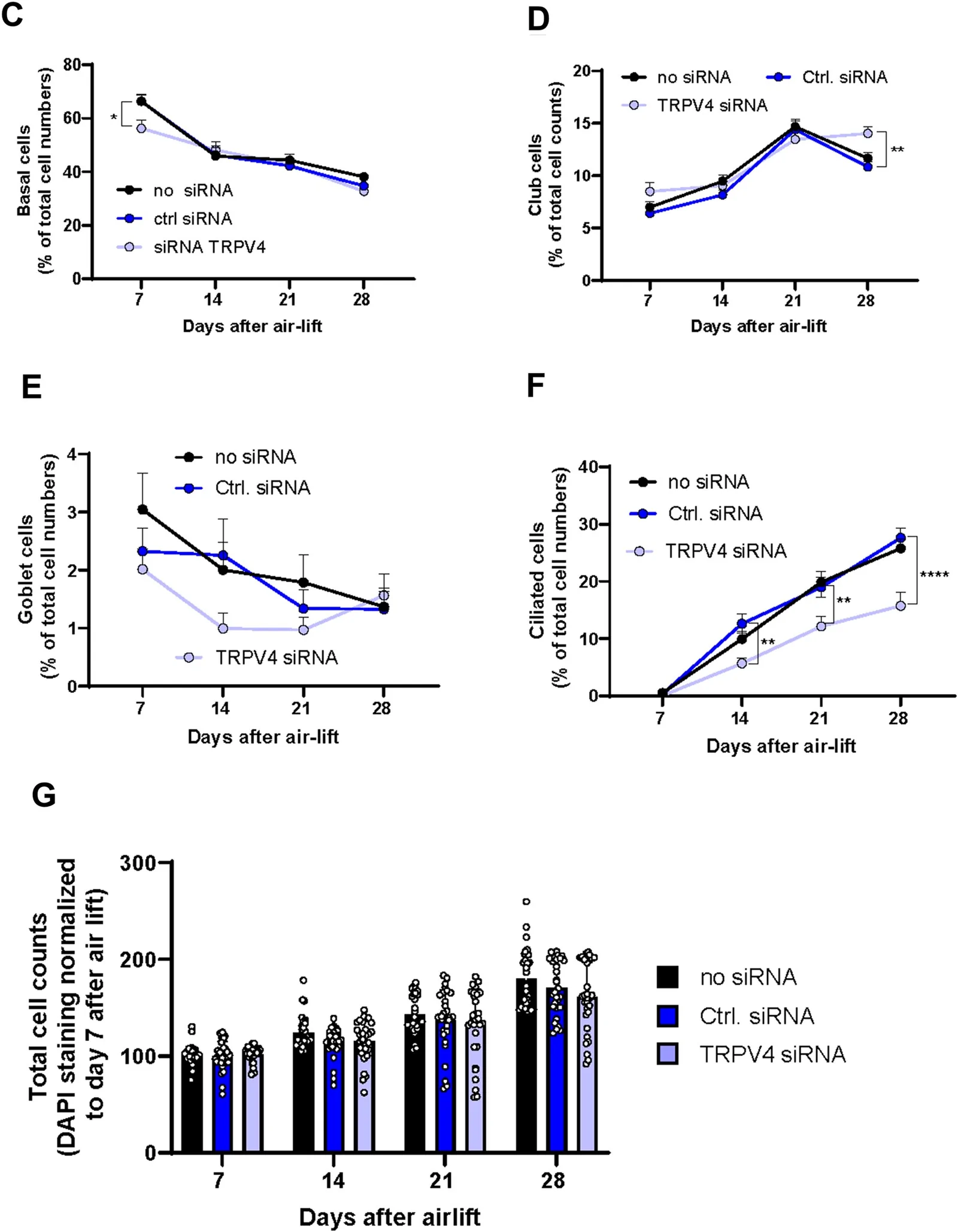
Immunofluorescence images and numbers of cells differentiated to a pseudostratified epithelium in an air-liquid interface (ALI) model with human bronchial epithelial cells (HBECs) with or without downregulation of transient receptor potential vanilloid 4 (TRPV4) channels. (A) 3D cross-section through a filter insert with differentiated cells stained with fluorescent-coupled antibodies against cell-specific proteins as indicated or all nuclei with DAPI (blue). (B) Top views on filter inserts 7 and 28 days after air lift. Naïve cells and cells transfected with control siRNAs (Ctrl. siRNA) or TRPV4-specific siRNAs (TRPV4 siRNA) are stained with fluorescent-coupled antibodies against cell specific proteins as indicated. (C) Percentage (%) of basal cells in total cell counts during differentiation in an ALI model of untransfected (no siRNA) HBECs and of cells transfected with Ctrl. siRNA or TRPV4 siRNA. (D) Percent (%) of club cells in total cell counts during differentiation in an ALI model of untransfected (no siRNA) HBECs and of cells transfected with Ctrl. siRNA or TRPV4 siRNA. (E) Percent (%) of goblet cells in total cell counts during differentiation in an ALI model of untransfected (no siRNA) HBECs and of cells transfected with Ctrl. siRNA or TRPV4 siRNA. (F) Percent (%) of ciliated cells in total cell counts during differentiation in an ALI model of untransfected (no siRNA) HBECs and of cells transfected with Ctrl. siRNA or TRPV4 siRNA. (G) Total cell counts identified by DAPI staining of nuclei counts during differentiation in an ALI model of untransfected (no siRNA) HBECs and of cells transfected with Ctrl. siRNA or TRPV4 siRNA. Data represent mean + SEM from 3 donors. Significance between means of cells transfected with control siRNA and TRPV4-specific siRNAs was analyzed using 2-way ANOVA: *P <.05, **P <.01, ****P <.0001.

In a translational approach, we established a human ALI model (Figure 5A) using HBECs^46^ from 3 healthy human donors (Table S1). The differentiation process to a pseudostratified epithelium was evident in cross-sections by an increasing thickness of the attached cell layer and the appearance of cilia on day 14 after the air lift (Figure 5B). We again detected no gross morphological differences in TRPV4^-/-^ cells compared to WT HBECs. As human basal cells next to ciliated cells express high amounts of TRPV4 mRNA^47^ (data published on www.ipfcellatlas.com), we set out to downregulate TRPV4 expression with specific siRNAs at an early time point (Transfection 1 with the initial seeding of basal cells on inserts 3 days before air lift) or a late time point (Transfection 2 after final differentiation to a pseudostratified epithelium 28 days after air lift) (Figure 5A). We reasoned that at the earlier time point we would be able to identify a role for TRPV4 channels in the differentiation of basal cells to SP-A and SP-D secreting club cells, and at the later time point we would test for TRPV4 function in SP-A and SP-D secretion to the mucous layer. By adding a TRPV4-specific siRNA with the transfection reagent to the medium in the upper compartment during the initial seeding of basal cells in inserts, we were able to suppress TRPV4 protein production by 93%, 89%, and 71% ± 4% on days 2, 5, and 10 after transfection, respectively (Figure S8A, B). TRPV4 protein production was also significantly downregulated 14, 21, and 28 days after transfection with a TRPV4-specific siRNA (Figure S8C). TRPV4 mRNA expression also increased during differentiation and was already not significantly different any more at day 28 (Figure S9). To test for functional expression of TRPV4 channels in human basal cells, we again performed Ca^2+^ imaging experiments with a TRPV4 activator (GSK 1016790A) and a TRPV4 inhibitor. While untransfected and control transfected cells showed a clear increase in intracellular Ca^2+^ levels after adding a TRPV4 channel activator (Figure S10A, B), which was abolished by a TRPV4 inhibitor (Figure S10B), TRPV4 siRNA-transfected cells showed no changes (Figure S10C). Next, we quantified cell numbers by using specific fluorescence-coupled antibodies (ab) for 4 cell types (Figure 6A, B). While numbers of club (marked by CC10 ab) and ciliated cells (marked by acetylated α-tubulin ab) increased during the differentiation process as described^48^^,^^49^ (Figure 6D, F), the amount of goblet cells (marked by MUC5AC ab) stayed at a low level (Figure 6E). We detected a lower number of basal cells at day 7 and a higher number of club cells at day 28 in cells transfected with the TRPV4-specific siRNAs compared to the control siRNAs. However, in clear contrast to the other cell types, numbers of ciliated cells were significantly decreased after downregulation of TRPV4 channels on days 14, 21, and 28 by a TRPV4-specific siRNA in comparison to control siRNAs added during seeding of basal cells to the filter inserts (Figure 6B, F). Along this line, acetylated α-tubulin levels normalized to control cells were also decreased after downregulation of TRPV4 on days 14 and 28 after air lift (Figure S11A), while total levels increased during basal cell differentiation (Figure S11B). Most interestingly, total cell counts (Figure 6G) and number of basal cells (Figure 6C) were not significantly different after downregulation of TRPV4 channels compared to controls at day 28 after air lift, pointing to a differentiation block of ciliated cells but not a complete loss of cells in the absence of TRPV4 protein.

**Figure 7:**
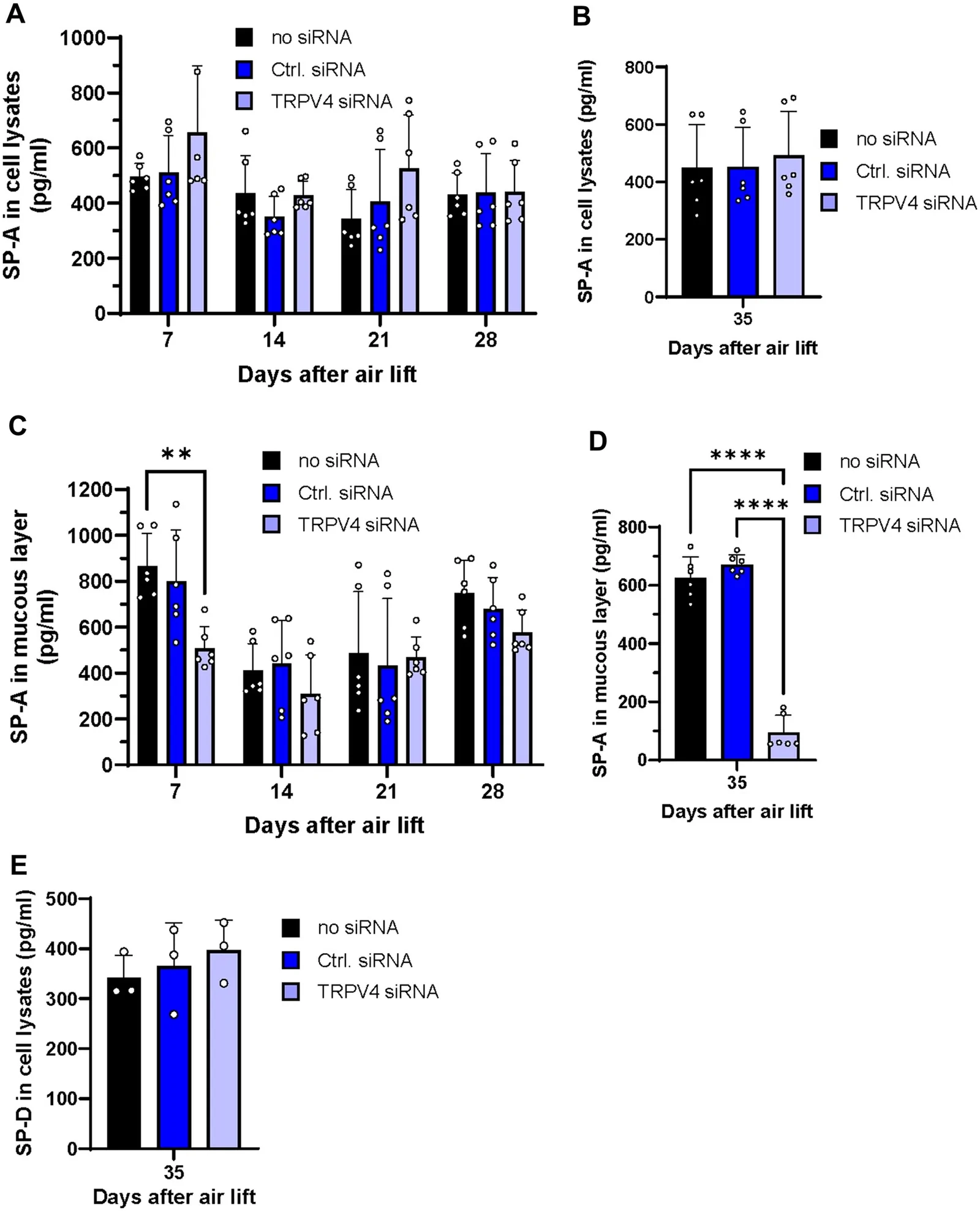
Surfactant protein A (SP-A) and D (SP-D) expression quantified by ELISA in an air-liquid interface (ALI) model with human bronchial epithelial cells (HBECs) in cell lysates and mucous layers after downregulation of transient receptor potential vanilloid 4 (TRPV4) channels. (A) SP-A expression in cell lysates from the ALI model 7, 14, 21, and 28 days after air lift in untransfected (no siRNA) HBECs and cells transfected with a control siRNA (Ctrl. siRNA) or a TRPV4-specific siRNA (TRPV4 siRNA) 3 days before air lift. (B) SP-A expression in cell lysates from the ALI model 35 days after air lift in untransfected (no siRNA) HBECs and cells transfected with Ctrl. siRNA or a TRPV4 siRNA at day 28 after air lift. (C) SP-A expression in the mucous layer of the ALI model 7, 14, 21, and 28 days after air lift in untransfected (no siRNA) HBECs and cells transfected with Ctrl. siRNA or a TRPV4 siRNA 3 days before air lift. (D) SP-A expression in the mucous layer of the ALI model 35 days after air lift in untransfected (no siRNA) HBECs and cells transfected with Ctrl. siRNA or TRPV4 siRNA at day 28 after air lift. (E) SP-D expression in cell lysates from the ALI model 35 days after air lift in untransfected (no siRNA) HBECs and cells transfected with Ctrl. siRNA or TRPV4 siRNA at day 28 after air lift. Data present mean + SEM from 3 different donors. Significance between means was analyzed using 2-way ANOVA: **P <.01, ****P <.0001.

As production of TRPV4 protein by siRNA-mediated downregulation partially and fully recovered on protein (Figure S8A-D) and mRNA level (Figure S9) 28 days after air lift, respectively, we chose an alternative later transfection time point to study the role of TRPV4 channels on SP-A and SP-D levels in differentiated HBECs 28 days after air lift (Figure 5A). TRPV4 channel protein expression was indeed significantly downregulated at day 35 after air lift after application of TRPV4-specfic siRNAs at this later time point (Figure S12A, B). While levels of both SPs were not different in cell lysates with downregulated TRPV4 channels (Figure 7A), SP-A was significantly reduced in mucous layers washed off from cell layers (Figure 7C) 7 days after air lift and recovered to normal levels 14 days after air lift. A successful downregulation of TRPV4 channels at a later time point (day 28; Figure S6A, B) was however again successful in reducing SP-A levels 35 days after airlift in the mucous layer (Figure 7D), but not in cell lysates (Figure 7B). While our ELISA was not sensitive enough to detect SP-D levels in mucous layers, SP-D levels in cell lysates at day 35 after air lift were again not significantly different (Figure 7E), similar to SP-A levels (Figure 7B). In contrast to murine AT2 and tracheal epithelial cells as well as HBECs, TRPV4 proteins were not detected in AT2 cell supernatants, murine, and human mucous layers (Figure S13A, B).

Thus, lower SP-A and SP-D levels in bronchial fluids are not due to lower cell numbers of club, nor to the production in these cells. Similar results were obtained from the TRPV4-deficient mouse model (Figures 1 and 2). Therefore, our findings provide evidence for an important role of TRPV4 channels in secretion of SP-A and SP-D in the pseudostratified epithelium.

## Discussion

TRPV4 channels are expressed in different tissues of the respiratory tract. They play an important role in fibroblast-to-myofibroblast differentiation during the progression of pulmonary fibrosis^35^ and endothelial barrier function for immune cell invasion (reviewed previously^50^). But their exact function in the lung alveolus and tracheobronchial epithelium is still elusive.^20^^,^^51^ We recently identified TRPV4 function in AT1 and AT2 cells of the lung alveolus.^39^ Ablation of TRPV4 channels resulted in a decreased production of pro-SP-C in AT2 and lower aquaporine 5 (AQP-5) expression and membrane localization in AT1 cells, which were most likely responsible for emphysema like changes in older mice and an increased ischemia-reperfusion–induced edema formation, respectively.^39^

Here, we quantified 2 other surfactant proteins, SP-A and SP-D, in the same TRPV4^-/-^ mouse model. In contrast to SP-B and SP-C, which closely interact with phospholipids and are integral members of the surfactant, SP-A and SP-D are secreted independent of lamellar bodies,^40^^,^^41^ bind to pathogens, and are essential for a strong innate immunity.^3^^,^^4^^,^^6^^,^^7^^,^^18^ Most interestingly, levels of both proteins were significantly reduced in lung lysates and BALF of TRPV4^-/-^ mice in comparison to WT controls (Figure 1A, B, E, F), while production was normal in AT2 cells in vitro (Figure 2D, E). However, SP-A and SP-D levels in the supernatant of TRPV4-deficient AT2 cells were also significantly decreased compared to WT cells (Figure 2F, G). As these data point to a possible function of TRPV4 channels in secretion of SP-A and -D from AT2 cells, we used 2 established assays to probe a possible direct physical interaction with TRPV4 channels. SP-A and -D were co-immunoprecipitated after overexpression in HEK293 cells with antibodies specific for TRPV4 and vice versa (Figure 3A-D), while TRPC6 channels failed to coimmunoprecipitate SP-A (Figure S5). Moreover, using a PLA, a close interaction of both SPs with TRPV4 channels was detected in primary AT2 cells from WT, but not from TRPV4^-/-^ mice (Figure 3E). However, this interaction was predominantly in inner cell compartments (Figure S7). Therefore, TRPV4 might be essential for secretion of SP-A and SP-D by physically interacting in cell organelles and supporting translocation of both SPs to the plasma membrane by a still to be identified molecular mechanism.

As SP-A and -D are also produced in the tracheal bronchial region by club cells,^43^^,^^44^ we utilized an ALI model to quantify protein levels in cell lysates of the pseudostratified epithelium and the mucous layer (Figure 4A, B). Again, production in epithelial cells was not changed in TRPV4^-/-^ cells compared to WT control cells, while protein levels in the mucous layer were significantly reduced (Figure 4C-F). In a translational approach, we obtained HBECs from 3 healthy donors to establish a human ALI model (Figure 5A, B) and downregulated expression levels of TRPV4 channels by specific siRNAs (Figure S8A, B). Similar to our results obtained in murine lung epithelial cells, SP-A and SP-D levels were not significantly changed in cell lysates, but SP-A levels were reduced in mucous layers (Figure 7A-D). Final numbers of club and goblet cells were not smaller, but differentiation of ciliated cells was reduced in the human ALI model (Figure 6B-F). The possible role of TRPV4 in differentiation of ciliated cells was a surprising, but interesting observation that warrants future investigations.

Reduced levels of SP-A and SP-D are clearly not due to a decreased number of AT2 cells in the murine alveolus^39^ and/or club cells in the human bronchial region (Figure 6B, 7B). However, as a secondary finding we identified a role for TRPV4 channels in human basal cell differentiation to ciliated cells, which were significantly reduced in cell numbers in the human ALI model (Figure 6B, F). As total cell numbers were not significantly different and the decrease of ciliated cells (Figure 6G) was not accompanied by a parallel large increase of progenitor cells including basal (Figure 6C) and club cells (Figure 6D), the absence of TRPV4 protein may not affect early commitment to the ciliated cell lineage but specifically affect differentiation to ciliated cells at a later stage prior to ciliogenesis.

In a previous study, TRPV4 channel expression was confirmed in murine ciliated cells and TRPV4^-/-^ mice had a reduced ciliary beat frequency induced by ATP in comparison to WT controls.^29^ In the human respiratory tract, however, a reduced number of ciliated cells is expected if TRPV4 channels are not functional (Figure 6F) and might compromise mucociliary clearance.

Loss of SP-A and SP-D was already studied in SP-A– and SP-D–deficient mouse models. While these mice show no major changes in surfactant activity and normal lung function,^52^^,^^53^ a complex immunological phenotype was apparent (previously reviewed^7^). SP-A^-/-^ animals are more susceptible for infections by, for example, *Streptococcus*^54^ and RSV,^55^ but also produced more TNF-α after application of LPS^56^ in comparison to WT controls. SP-D–deficient mice showed an increased number of activated foamy alveolar macrophages with high levels of reactive oxygen species and matrix metalloproteinases, which might be responsible for an unprovoked progressive postnatal inflammation and the development of emphysema.^53^^,^^57^ SP-A/SP-D double-deficient mice mainly resemble the lung phenotype seen in SP-D^-/-^ mice.^58^ We already described an emphysema like phenotype of the TRPV4^-/-^ mouse model,^39^ which was also apparent in SP-C–deficient animals.^59^

Surfactant secretion in producing cells is strongly dependent on intracellular Ca^2+^ levels.^60^ Some authors propose TRPV2 channels as the main source of Ca^2+^ influx into the cells, which was blocked by ruthenium red.^61^ But next to TRPV2, ruthenium red is also a potent blocker of TRPV4 channels, which can be activated by stretch and changes in osmolarity in the respiratory tract. However, as physical interaction occurs mostly in intracellular compartments (Figure S7) and not at the plasma membrane, a mechanosensitive activation of TRPV4 channels at and Ca^2+^ influx near the plasma membrane is less likely. While we at this point can only speculate about an intracellular interaction of TRPV4 with SP-A and -D proteins in secretory vesicles of AT2 cells, a location and function of TRPV4 in organelles like mitochondria,^62^ nuclei,^63^ and the ER^64^ was already reported. Splice variants of TRPV4 are specifically retained in the ER,^64^ and an Os9 protein interacts with the TRPV4 N-terminus to reduce plasma protein expression of this channel.^65^ Future studies need to further analyze the regulation of SP-A as well as SP-D secretion by TRPV4 channels in intracellular compartments of AT2 and club cells.

In summary, our data highlight novel essential functions of TRPV4 channels in SP-A and SP-D secretion. As outlined in the introduction, both SPs are linked to numerous immunological and pathophysiological processes in the respiratory tract including lung repair, asthma, IPF, and inflammation (reviewed previously^66^). Therefore, this TRPV4-deficient mouse model with reduced SP-A and SP-D secretion might not only result in a defective immune response against pathogens, but also in changes in asthma and IPF progression as well as lung repair and inflammation, which needs to be identified in future studies.

## Supplementary Material

aanag023_Supplementary_Data

## Data Availability

This article has a data supplement, which is accessible at the Supplements tab. The data are available from the authors upon reasonable request.
