# Supplementary material for: Transient receptor potential vanilloid 4 (TRPV4) channels mediate pulmonary surfactant protein A and D secretion
Source: Am J Respir Cell Mol Biol. 2026 Feb 21;74(7):896–910. doi: 10.1093/ajrcmb/aanag023 (PMC13316927; doi:10.1093/ajrcmb/aanag023)
Supplement: aanag023_Supplementary_Data [file aanag023_supplementary_data.zip › aanag023_Supplementary_Data/SupplementAlt260312.pdf]

Transient receptor potential vanilloid 4 (TRPV4) channels mediate pulmonary surfactant protein A and D secretion

Philipp Alt[1], Isabel Müller[1,5], Martina Kiefmann[1,5], Thomas Gudermann[1,5], Wolfgang M. Kuebler[2], Matthias Griese[3,5], Claudia A. Staab-Weijnitz[4,5], and Alexander Dietrich[1,5]

[1]Walther Straub Institute of Pharmacology and Toxicology, Medical Faculty, LMU-Munich, Munich Germany.

[2]Institute of Physiology, Charité - Universitätsmedizin Berlin, Berlin, Germany

[3]Department of Pediatrics, Dr. von Hauner Children's Hospital, University Hospital, LMU-Munich, Munich, Germany

[4]University of Colorado, Anschutz Medical Campus, Department of Pediatrics and Division of Pulmonary Allergy and Critical Care Medicine, School of Medicine, Aurora, CO, USA

[5]Deutsches Zentrum für Lungenforschung (DZL), CPC-M, Munich, Germany

Online data Supplement

Supplementary Material and Methods

Reagents and Antibodies

For the experiments, following reagents and antibodies were used: Pierce BCA Assay (Thermo Fisher Scientific, Waltham, Massachusetts, USA, #23225), PneumaCult™ Ex Plus Kit (Stemcell Technologies, Vancouver, Canada, #05040), PneumaCult™ ALI-Medium Kit (Stemcell Technologies, Vancouver, Canada, #05021), AlexaFluor®488 anti-CC10 antibody (Santa Cruz, Dallas, Texas, USA), AlexaFluor®488 anti-p63 antibody (abcam, Cambridge, UK), AlexaFluor®555 anti-mucin5ac antibody (abcam, Cambridge, UK), AlexaFluor®647 anti-α-tubulin antibody (abcam, Cambridge, UK), anti-TRPV4 antibody (Merck, Darmstadt, Germany), anti-vinculin antibody (Sigma Aldrich (Merck), St. Louis, Missouri, USA),

Primary culture of murine AT2 cells and murine ALI model

Alveolar epithelial Type 2 cells (AT2) were isolated from wild type or TRPV4-/- mice as previously described (1). In brief, 4-5 mice were euthanized by cervical dislocation and chest was opened. A cannula was installed into the trachea. The lungs were flushed with 10 mL NaCl (Braun AG, Melsungen, Germany, #3200950) via the right ventricle of the heart and 1 mL dispase solution (Thermo Fisher Scientific, Waltham, Massachusetts, USA, #354235) as well as 300 µL of a 1% low melting agarose (Sigma Aldrich, St. Louis, Missouri, USA, #A9414) solution was instilled via the trachea. After solidifying, whole lungs were cut out and stored in 1 mL dispase solution for 45 min at room temperature. Lungs were minced, and passed through different nylon filter membranes (100µm, 20µm, 10µm; Sefar, Heiden, Switzerland, #3A03-0100-115-01, #3A3-0020-102-10, #3A3-0010-102-00) to get single cell suspension and cells from all lungs were pooled. Collected cells were equally distributed on CD16/32/45 antibody (BD Biosciences, Meylan, France, #553142, #553076) coated petri dishes and incubated for 30 min at 37 °C to remove hematopoietic cells. Supernatants were transferred to TC-dishes and incubated for 60 min at 37 °C to remove fibroblasts. Supernatant was collected and, after centrifugation (400 x g, 5 min, 15 °C), cells were seeded and incubated in 6-well plates. Depending on the desired phenotype for the experiment, cells were cultured for 24h and 48h after isolation.

Murine tracheal epithelial cells (mTEC) were isolated either from wildtype or TRPV4-/- mice as described in former protocols (Eenjes et al. (2018); (You & Brody, 2013)). Mice were euthanized by cervical dislocation, tracheas were cut out and excess tissue was removed using a binocular. Tracheas were incubated in pronase (Merck, Darmstadt, Germany, #10165921001) solution (0,12% W/V in Ham´s F12) in Falcon tubes over night at 4 °C. The next day, fetal bovine serum (FCS) (Thermo Fisher Scientific,

Waltham, Massachusetts, USA, #26140079) was added to stop enzymatic digestion and tubes were inverted 15-20 x. The tracheas were put into another Falcon tube with 3 mL Ham´s F12 (Lonza, Basel, Switzerland, #BE12-615F)/10 % FCS and the procedure was repeated twice. Collected cell suspensions were centrifuged at 500 x g at 4 °C for 10 min. Supernatant was discarded, the cell pellet was resuspended in DNAse (PanReacAppliChem, #A3778) solution (0,5 mg/mL in Ham´s F12 with 1 mg/mL bovine serum albumin (BSA, Thermo Fisher Scientific, USA, #30036578) and put on ice for 5 min. After another centrifugation step (500 x g at 4 °C for 10 min), the pellet was resuspended in mTEC/Basic medium and cells were seeded onto Primaria™ cell culture dishes (BD Bioscience, #10741392). After 3-4 h in the incubator, fibroblasts attach to the dishes and supernatant with non-attached cells was collected, centrifuged and resuspended in mTEC/Plus medium. After counting, cells were seeded on collagen-coated (rat tail collagen Type I, Sigma Aldrich (Merck), USA, #C3867) 12-well Transwell™-inserts (Merck, Germany, #CLS3460) at 37 °C and 5 % CO2 and were kept for 3 days with mTEC-Medium - basal and apical - until confluence was reached. After that, air-lift was performed by removing apical medium and changing basal medium to mTEC-Basic medium supplemented with 2 % V/V Nu-Serum® (Corning (Merck), Germany, #10632702). Cells were differentiated for 21 days.

Ca^{2+} Imaging of AT2 and basal cells

AT2-cells or basal human bronchial epithelial cells (HBECs) were grown on either 24mm (AT2) or 12mm (NHBECs, Collagen-coated) coverslips. AT2-cells were used 36-48h after isolation, to ensure AT2-characteristic cells. Lysotracker™ Green DND 26 (Invitrogen, Thermo Fisher Scientific, #L7526), which stains acidic lamellar bodies, was applied to confirm an AT2-cell type (2, 3) . The human donor cells were incubated with control siRNA or specific siRNA against TRPV4 or no RNA as described in methods

part for siRNA-knockdown experiments and were used on day 3 after initial transfection. On the day of measurement, cells were loaded with 10µM Fura-2 AM (Invitrogen, Thermo Fisher Scientific, #F1221) in HBSS (containing Ca^{2+} and Mg $^{2+}$) for AT2-cells and 4µM Fura-2-AM in HBSS for HBECs for 40min at 37°C. After incubation, coverslips were washed with HBSS and placed into a quick-change chamber (Warner Instruments, Holliston, USA) with 450µL (24mm) or 150µL (12mm) of HBSS. Imaging was performed with a 40x oil-objective of a Leica DM98 fluorescence microscope. Intracellular Ca^{2+} concentration was measured following the application of TRPV4-activator (GSK10160970A, Merck, # 5.30533, 100nm (AT2), 100µM or 300µM (HBECs)) with or without using TRPV4-blocker (HC067047, Merck, # 616521). Cells were excited at 340 and 380 nm and fluorescence emissions were recorded at 510 nm. Emission ratios at wave lengths of 340nm and 380nm were calculated.

Bronchoalveolar Lavage (BAL)

To investigate the amounts of surfactant protein in secreted fractions, lungs of wildtype and TRPV4-/- mice after euthanization were instilled 3 x with 500µL PBS (Sigma Aldrich, USA, #D8537) supplemented with phosphatase and protease inhibitor) via the trachea and gently withdrawn. Lavage was collected, pooled and centrifuged at 4 °C and 10.000 rpm for 5 min. Supernatants were concentrated 12 times via Amicon Ultracel 10K centrifuge filters (Merck, Germany, #UFC501024) and used for protein analysis. Protein samples. As reference, body weight and total lung volume (deep inflation) of the mice were measured using a flexivent (Scireq, Canada) before collecting BAL.

Immunofluorescence staining and confocal imaging.

NHBECs and mTECs were washed with cold PBS, fixed in 4% PFA/PBS (15 min at RT) on inserts and then washed thrice with PBS. Cells were permeabilized for 15-30

min (depending on time point of differentiation) with 0.5 % Triton X100 solution in PBS and then washed 3 x 5 min with PBS. Blocking solution containing 4 % BSA in PBS was applied for 1 h (RT) and inserts were incubated at 4 °C overnight with primary antibodies (dilution in 2 % BSA in PBS; basal cell marker p63, Abcam, UK, #ab246727, 1:200; club cell marker CC10, Santa Cruz Biotech, Dallas, Texas, USA, #sc-365992AF488, 1:200; goblet cell marker MUC5AC, abcam, UK, #ab218714, 1:200; ciliated cell marker acetyl-Tubulin, abcam, UK, #ab218591, 1:500; SP-A antibody, abcam, UK, #115791, 1:1.000; SP-D antibody, R&D Bio-Techne, France, 1:1.000). The next day, inserts were washed 3 x 5 min with 0.1 % BSA in PBS. Nuclei staining was performed using DAPI solution (0.1 mg/L in PBS) for 10 min at RT. Inserts were washed again 2 x 5 min with 0.1 % BSA in PBS and finally 2 x 5 min with PBS. Inserts were cut out, mounted on glass cover slides (Epredia, New Erie Scientifice, Netherlands, #J1800AMNZ) with mounting medium (ProLong™ Glass Antifade Mountant, Thermo Fisher Scientific, USA, #P36980) and let dry at RT overnight. Slides were stored at 4 °C if not immediately used for imaging. We used fluorescent dye coupled antibodies for the experiments with different wavelengths (488 nm for p63 and CC10, 555 nm for MUC5AC, 647 nm for acetyl-tubulin). Confocal images were taken as z-stacks with a Zeiss LSM 880 microscope using ZEN Black software (version 2.3). Image processing was done using ZEN Blue software (version 3.4, Carl Zeiss, Jena, Germany) and Fiji software (Image J v. 1.53c, Wayne Rasband, NIH, USA). Stacks were separated with respective channels (405 nm, 488 nm, 555 nm, 647 nm) and composed images were generated. Counting of cells was done with Fiji software. For analyses, every insert was imaged 10 x on different areas across the slide with a 20x air objective. For each time point, there were two replicates for each condition and experiments were performed with three different healthy donors.

SDS-PAGE and Western Blot analysis

The expression of proteins was determined by Western blot analysis, as previously described (4). Cells were lysed with 150 µL RIPA buffer (containing phosphatase and protease inhibitors) and placed on ice for 30 min. Protein concentration was measured with the Pierce BCA Protein Assay Kit (Thermo Fisher Scientific, #23225) according to manufacturer´s protocol. The protein samples were mixed with 5 x laemmli buffer (3 mL 2.6 M TRIS/HCl pH 6.8, 10 mL glycerol, 2 g SDS, 2 mg bromophenol blue, 5 mL β-mercaptoethanol) and heated for 10 min at 95 °C. Then, samples with 20 µg of total protein were loaded onto an SDS-PAGE gel (stacking gel 4% polyacrylamide, separating gel 10 % polyacrylamide) and electrophoresis was run for 30 min at 70 V at separating gel and then for 100 min at 100 V. Proteins then were transferred from the gel to a Roti®-PVDF membrane (Carl Roth, Karlsruhe, Germany, #T830.1) in a wet-transfer system from BioRad (Feldkirchen, Germany) with 90 mA for 16 h at 4 °C. Blocking was performed using 5 % low-fat milk powder (Carl Roth, #T145.2) in TBS-T (0.1 % TWEEN 20 in TBS buffer) for 1 h at RT. All antibodies were diluted according to supplier´s datasheet in blocking solution (SP-A antibody, abcam, UK, #115791, 1:1.000; SP-D antibody, R&D Bio-Techne, France, 1:1.000; TRPV4-antibody, Merck, Germany, #MABS466, 1:10.000; Vinculin-antibody, Sigma Aldrich, USA, #V9131, 1:10.000; anti-ß-actin-POX, Merck, Germany, #A3854, 1:10.000; anti-mouse-IgG-HRP, Cell Signaling, Danvers, Massachusetts, USA, #7076, 1:2.000; anti-rabbit-IgG-POX, Sigma Aldrich, USA, #A6151, 1:10.000; anti-goat-IgG-POX, Sigma Aldrich, USA, #A5420, 1:10.000). Membranes were incubated in primary antibodies solution overnight (16 h) at 4 °C, washed 3 x 5 min with TBS-T and incubated again with peroxidase-conjugated secondary antibody solutions for 2 h at RT. After washing again 3 x 5 min with TBS-T, membranes were incubated with SuperSignal West Pico or Femto (Thermo Fisher Scientific, #34577) sensitivity substrates and imaged using an Odyssey-Fc unit (Licor, Lincoln, NE, USA).

Co-Immunoprecipitation

Possible interactions of Surfactant Protein A and D with TRPV4 were investigated using a HEK293T-cell overexpression system. After transfection of HEK cells with plasmids coding for SP-A (FLAG-tagged) and TRPV4 tagged with eGFP (or SP-D (FLAG-tagged) and TRPV4eGFP) with a mix of Opti-MEM Reduced-Serum Medium, TransIT®-2020 Transfection reagent and 2.5 µg of Plasmid DNA (Origene, USA, #MR201760, #MR205806), cell lysates were collected 48h after initial plasmid transfection. Lysates were centrifuged and supernatant was incubated with Dynabeads® (ThermoFisher Scientific, USA, #10001D) coated with anti-FLAG-ab (Origene, USA, #TA50011-100-OR) or TRPV4-ab. After separation via MagnaRack™ and multiple washing steps, proteins captured with beads were eluted with a low pH Glycine-buffer and analyzed via Western Blot.

Enzyme-linked immunosorbent assay (ELISA)

For samples in NHBEC-ALI-culture, classical Sandwich-ELISA was used according to manufacturer´s protocol (Novus Biotech, USA, Human-surfactant protein A ELISA-kit, #NBP2-76692). In brief, standard dilution or sample was applied to anti-human SP-A antibody pre-coated 96-well plates and incubated for 2 hours at 37°C. A biotinylated detection antibody was added and after 60 min incubation at 37 °C, wells were washed thrice with supplied washing buffer. An Avidin-HRP-conjugate was added and incubated for 30 min at 37°C and wells were washed again five times. After adding the substrate reagent solution and incubating again for 15 min at 37 °C, stop solution was applied to each well and optical density was measured immediately at 450 nm. Amount of surfactant protein in the respective sample was calculated according to the standard curve generated in the same run. ELISA for surfactant Protein D detection

(Quantikine® ELISA, R&D Bio-Techne, France, #DSFPD0; Human Surfactant Protein D/SP-D ELISA Kit, Abcam, UK, #AB239431-1001) was carried out with similar steps, with a longer initial incubation (3 h) and was completely performed at room temperature. Samples from supernatant of AT2-cell culture were measured using Aliquots from centrifuged cell culture supernatant on day 2 after isolation of primary AT2 cells. ELISA-steps were carried out as described above for Surfactant Protein A (Novus Biotech, USA, Mouse Surfactant Protein A ELISA Kit (Colorimetric) , #NBP2-76693) and Surfactant Protein D (Quantikine™ ELISA, R&D Systems, USA, #MSFPD0).

Quantitative reverse transcription PCR (qRT-PCR)

Gene-expression of collected samples was measured with quantitative Reverse-Transcriptase Polymerase Chain Reaction (qRT-PCR). Therefore, RNA-Lysates were collected and RNA was isolated via RNeasy® Plus Mini-Isolation Kit (Qiagen, Germany, #74136) according to manufacturer´s protocol. Then, 1 µg of RNA was transcribed into cDNA with RevertAid H Minus First Strand cDNA Synthesis Kit (Thermo Fisher Scientific, USA, #K1632). Quantitative qPCR was performed using dilution of cDNA with specific Primers for genes of interest (sequence see Table E4) and ABsolute qPCR SYBR Green Mix (Thermo Fisher Scientific, USA, #AB-1285/B) in a 96 well-plate and was analyzed with a LightCycler480 from Roche. Received Ct-values were taken for calculation of expression of the respective genes normalized to housekeeping gene ß-actin. Normalized values of samples were then referred to ctrl samples for detection of possible differences between ctrl, ctrlRNA or siRNA treated samples.

References:

1. Weber J, Rajan S, Schremmer C, Chao YK, Krasteva-Christ G, Kannler M, et al. TRPV4 channels are essential for alveolar epithelial barrier function as protection from lung edema. *JCI Insight.* 2020;5(20).
2. Van der Velden JL, Bertoncello I, and McQualter JL. LysoTracker is a marker of differentiated alveolar type II cells. *Respiratory research.* 2013;14(1):123.
3. Bove PF, Grubb BR, Okada SF, Ribeiro CM, Rogers TD, Randell SH, et al. Human alveolar type II cells secrete and absorb liquid in response to local nucleotide signaling. *J Biol Chem.* 2010;285(45):34939-49.
4. Hofmann K, Fiedler S, Vierkotten S, Weber J, Klee S, Jia J, et al. Classical transient receptor potential 6 (TRPC6) channels support myofibroblast differentiation and development of experimental pulmonary fibrosis. *Biochim Biophys Acta.* 2017;1863(2):560-8.

Supplementary Tables

Table S1. List of human donors of human bronchial epithelial cells (HBEC) used in ALI

Number	Sex	Age	Race	Health condition	Smoker	Alcohol use
**18TL190281**	Male	75	B	Non-diseased	No	No
**21TL347555**	Male	55	C	Non-diseased	No	No
**23TL240134**	Male	47	H	Non-diseased	No	No

Table S2. RNA-sequences of the Accell Human TRPV4 siRNA – SMARTpool

Target Sequence	Molecular weight (g/M)	Extinction coefficient
**GACUUGUUCAUGAAGAAAU**	13.492,7	364,722
**UUGUUACCAAGAUGUACGA**	13.508,7	364,188
**CUAUCCUCUUUGACAUCGU**	13.618,9	365,256
**UCUUCAUGAUCGGCUACGC**	13.589,8	347,456

Table S3. RNA-sequences of the Accell non-targeting pool

Target Sequence	Molecular weight (g/M)	Extinction coefficient
**UGGUUUACAUGUCGACUAA**	n.a.	n.a.
**UGGUUUACAUGUUUUCUGA**	n.a.	n.a.
**UGGUUUACAUGUUUUCCUA**	n.a.	n.a.
**UGGUUUACAUGUUGUGUGA**	n.a.	n.a.

Table S4. Primer sequences for quantitative reverse transcription (qRT) PCR of genes of interest and a housekeeping gene

Gene of interest	Forward	Reverse	PCR product (bp)
**TRPV4**	GCT CTT CAC TGG GGT CCT G	GCT GGA AGG AGC CAT CAA TG	101
**β-actin**	CCA ACC GCG AGA AGA TGA	CCA GAG GCG TAC AGG GAT AG	97

Table S5. Antibodies used for the Proximity Ligation Assay (PLA)

Primary antibodies			Secondaries
**Target**	**Host species**	**Ref. No.**	**PLA®-Probe**
**TRPV4**	mouse	Merck #MABS466	mouse MINUS (#DUO92004)
**SP-A**	rat	Abcam #*ab115791*	rabbit PLUS (#DUO92002)
**SP-D**	goat	R&D #AF1920	goat PLUS (#DUO92003)

Samples incubated with TRPV4/SP-A (mouse/rabbit) or TRPV4/SP-D (mouse/goat) and processed with anti-mouse-MINUS/anti-rabbit/PLUS or anti-mouse-MINUS/anti-goat-PLUS

Supplemental Figures

Supplemental Figure S1. Sex specific surfactant protein A (SP-A) and surfactant protein D (SP-D) levels in lung lysates and BAL fluid of WT and TRPV4-/- mice. (*A*) SP-A levels in lung lysates from male and female WT mice. (*B*) SP-A levels in lung lysates from male and female TRPV4-/- mice. (C) SP-D levels in lung lysates from male and female WT mice. (*D*) SP-D levels in lung lysates from male and female TRPV4-/- mice (*E*) SP-A levels in BAL fluids from male and female WT mice. (*F*) SP-A levels in BAL fluids from male and female TRPV4-/- mice. (*G*) SP-D levels in BAL fluids from male and female WT mice. (H) SP-D levels in BAL fluids from male and TRPV4-/- mice. Data present means + SEM from at least 4 mice. No significance between means was detected by using student's T-test.

Supplemental Figure S2. Sex specific surfactant protein A (SP-A) and surfactant protein D (SP-D) levels in lung lysates and BAL fluid of WT and TRPV4-/- mice. (*A*) SP-A levels in lung lysates from male WT and TRPV4-/- mice. (*B*) SP-A levels in lung lysates from female WT and TRPV4-/- mice. (*C*) SP-D levels in lung lysates from male WT and TRPV4-/- mice. (*D*) SP-D levels in lung lysates from female WT and TRPV4-/- mice (*E*) SP-A levels in BAL fluids from male WT and TRPV4-/- mice. (*F*) SP-A levels in BAL fluids from female WT and TRPV4-/- mice. (*G*) SP-D levels in BAL fluids from male WT and TRPV4-/- mice. (*H*) SP-D levels in BAL fluids from female WT and TRPV4-/- mice. Data present means + SEM from at least 4 mice. Significance between means was analyzed using student's T-test and is indicated as ** for $p<0.01$ and * for $p<0.05$.

Supplemental Figure S3. Ca^{2+} imaging of murine alveolar type 2 (AT2) cells after application of a TRPV4 activator with or without preincubation with an inhibitor. (*A)* differentiated AT2 cells were identified by staining their lamellar bodies with LysoTracker DND 26 and loaded with the Ca^{2+} indicator Fura-2 AM. Fluorescence ratios were analyzed at excitation wavelengths of 340nm and 380nm. (*B*) a TRPV4 channel activator (GSK: GSK 1016790A) was applied at the indicated time point. (*C*) a TRPV4 channel inhibitor (HC0974047) was applied 45 minutes before starting the analysis and a TRPV4 channel activator (GSK: GSK 1016790A) was applied at the indicted time point. ATP was added as a positive control. Data present means +/- SD for at least 7 cells in one representative analysis out of three experiments.

Supplemental Figure S4. Validation of TRPV4 expression in WT and TRPV4-/- mice. TRPV4 expression in cell lysates from AT2 cells 2 days after isolation. Western Blot showing three samples from each genotype representing five mice per sample (total of 15 mice each genotype). The upper part was stained with a TRPV4-specific antibody, which detects protein bands of the predicted size of TRPV4 proteins in lane 1-3. The lower part was stained with a specific β-actin antibody as loading control.

Supplemental Figure S5. Surfactant protein A (SP-A) and D (SP-D) do not physically interact with TRPC6 channels in a heterologous overexpression system. HEK293 cells were transfected with SP-A flag (SP-A), TRPC6 (TRPC6), both (SP-A/TRPV4) cDNAs or mock (-). Proteins were co-immunoprecipitated with TRPC6 or flag antibody, separated by PAA gel electrophoresis and identified in a Western Blot by (*A*) flag antibodies (Flag ab.) or (*B*) TRPC6 antibodies (TRPC6 ab.).

Supplemental Figure S6. Detection of SP-A and SP-D by specific antibodies in primary AT2 cells from WT and TRPV4-/- mice. Fluorescence images of AT2 cells from wild-type (WT) and TRPV4-deficient (TRPV4-/-) mice incubated with specific antibodies directed against SP-A (green fluorescence) or SP-D (red fluorescence) bound to fluorescence-coupled secondary antibodies. Nuclei were stained with DAPI (blue).

Supplemental Figure S7. Cellular localization of fluorescent signals formed by a Proximity Ligation Assay (PLA) with specific antibodies directed against TRPV4 channels and surfactant protein A (SP-A) or surfactant protein D (SP-D). (*A*) Fluorescence images of AT2 cells from wild-type mice incubated with specific antibodies directed against E-Cadherin and (*B*) after performing a PLA with antibodies directed against SP-A and TRPV4 channels. (C) Fluorescence images of AT2 cells from wild-type mice incubated with specific antibodies directed E-Cadherin and (*D*) after performing a PLA with antibodies directed against SP-D and TRPV4 channels. Red dots indicate interactions of TRPV4 channels with SP-A or –D. Cell nuclei were stained with DAPI in blue.

Supplemental Figure S8. Downregulation of TRPV4 channels in an Air-Liquid-Interface (ALI) model with human bronchial epithelial cells (HBEC). (*A*) Expression of TRPV4 proteins in cells transfected with control siRNAs (Ctrl.) or TRPV4 specific siRNAs (TRPV4) was quantified by Western Blotting of protein lysates of cells differentiated for the indicated time points using a TRPV4 specific antibody. β-actin served as loading control. (*B*) Quantification of TRPV4 expression after transfection of cells with control siRNAs (Ctrl. siRNA) or TRPV4 specific siRNAs (TRPV4 siRNA). Data represent means + SEM from 3 donors. Significance between means of each day

was analyzed using two-tailed unpaired Student's t-test and indicated as * for $p<0.05$. (*C*) TRPV4 expression in cell lysates from the ALI model 14, 21 and 28 days after air-lift in untransfected (no siRNA) HBEC and cells transfected with a control siRNA (Ctrl. siRNA) or a TRPV4 specific siRNA (TRPV4 siRNA) at day 3 before air lift in % of the untransfected controls. Data present means + SEM from 3 different donors with technical duplicates. Significance between means was analyzed using two-way ANOVA and is indicated as *** for $p<0.001$, ** for $p<0.01$ and * for $p<0.05$.

Supplemental Figure S9. Downregulation of TRPV4 mRNA in an Air-Liquid-Interface (ALI) model with human bronchial epithelial cells (HBEC). TRPV4 mRNA expression in cell lysates from the ALI model 7, 14, 21 and 28 days after air-lift in untransfected (no siRNA) HBEC and cells transfected with a control siRNA (Ctrl. siRNA) or a TRPV4 specific siRNA (TRPV4 siRNA) at day 3 before air lift in % of the untransfected controls Data present means + SEM from 3 different donors. Significance between means was analyzed using two-way ANOVA and is indicated as **** for $p<0.0001$, *** for $p<0.001$, ** for $p<0.01$ and * for $p<0.05$.

Supplemental Figure S10. Ca^{2+} imaging of human bronchial epithelial cells (HBEC) with or without downregulation of TRPV4 channels after application of a TRPV4 activator and/or inhibitor. Untransfected HBEC ((A) no siRNA) and cells transfected with a control siRNA ((B) Ctrl. siRNA) or a TRPV4 specific siRNA ((C) TRPV4 siRNA) were loaded with the Ca2+ indicator Fura-2 AM and fluorescence ratios were analyzed at excitation wave lengths of 340nm and 380nm. A TRPV4 activator (GSK 1016790A) and/or an TRPV4 inhibitor (HC097047) were applied at the indicated time points. Ionomycin (2 µM) was added as a positive control. Data present means +/- SD for at least 20 cells.

Supplemental Figure S11. Expression of acetylated α-tubulin in an Air-Liquid-Interface (ALI) model with human bronchial epithelial cells (HBEC) after down-regulation of TRPV4 channels. (*A*) Expression of acetylated α-tubulin in cell lysates from the ALI model 14, 21 and 28 days after air-lift in untransfected (No siRNA) HBEC and cells transfected with a control siRNA (Ctrl. siRNA) or a TRPV4 specific siRNA (TRPV4 siRNA) 3 days before air lift in % of untransfected controls. (*B*) Expression of acetylated α-tubulin in cell lysates from the ALI model 14, 21 and 28 days after air-lift in untransfected (No siRNA) hBEC and cells transfected with a control siRNA (Ctrl. siRNA) or a TRPV4 specific siRNA (TRPV4 siRNA) 3 days before air lift in % expression at day 14 after air-lift. Data present means + SEM from 3 different donors. Significance between means was analyzed using two-way ANOVA and is indicated as **** for $p<0.0001$ and ** for $p<0.01$.

Supplemental Figure S12. Downregulation of TRPV4 channels in an Air-Liquid-Interface (ALI) model with human bronchial epithelial cells (HBEC) at day 35 after air-lift. (*A*) TRPV4 protein expression in cell lysates from the ALI model at day 35 after air-lift in HBEC transfected with a control siRNA (Ctrl. siRNA) or a TRPV4 specific siRNA (TRPV4 siRNA) 28 days after air lift in % of Ctrl. siRNA transfected cells. (*B*) Representative Western Blot of TRPV4 protein expression in cell lysates from the ALI model at day 35 after air-lift transfected with a control siRNA (Ctrl. siRNA) or a TRPV4 specific siRNA (TRPV4 siRNA) 28 days after air lift incubated with a TRPV4 specific antibody. Data present means + SEM from 3 different donors. Significance between means was analyzed using two-tailed unpaired Student's t-test and is indicated as **** for $p<0.0001$.

Supplemental Figure S13. Validation of TRPV4 channel expression in cell lysates, but not in the broncheoalveolar lavage (BAL) and mucous layers. (*A*)

TRPV4 expression in cell lysates from wild-type (WT) and TRPV4-deficient (TRPV4-/-) AT2 cells (AT2) and broncheoalveolar lavages (BAL). (*B*) TRPV4 expression in cell lysates from wild-type (WT) and TRPV4-deficient (TRPV4-/-) murine tracheal epithelial cells (mTEC) as well as in human bronchial epithelial cells (HBECs) transfected with a control or TRPV4 specific siRNA and mucous layers. The upper part was stained with a TRPV4-specific antibody, which detects protein bands of the predicted size of TRPV4 proteins in lane 1-3. The lower part was stained with a specific β-actin antibody as loading control.

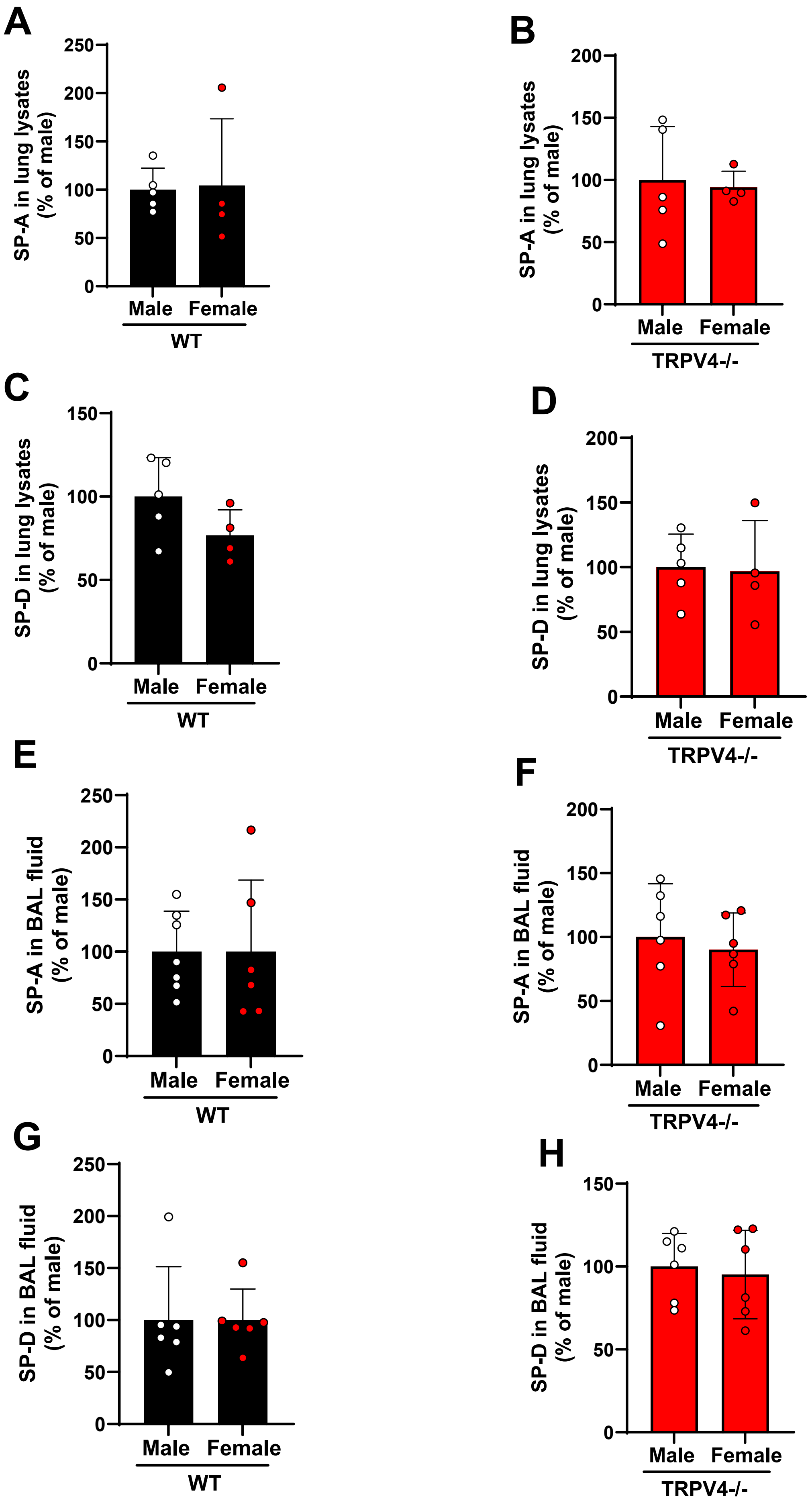

Supplemental Figure S1. Sex specific surfactant protein A (SP-A) and surfactant protein D (SP-D) levels in lung lysates and BAL fluid of WT and TRPV4-/- mice. (*A*) SP-A levels in lung lysates from male and female WT mice. (*B*) SP-A levels in lung lysates from male and female TRPV4-/- mice. (*C*) SP-D levels in lung lysates from male and female WT mice. (*D*) SP-D levels in lung lysates from male and female TRPV4-/- mice (*E*) SP-A levels in BAL fluids from male and female WT mice. (*F*) SP-A levels in BAL fluids from male and female TRPV4-/- mice. (*G*) SP-D levels in BAL fluids from male and female WT mice. (*H*) SP-D levels in BAL fluids from male and TRPV4-/- mice. Data present means + SEM from at least 4 mice. No significance between means was detected by using student's T-test.

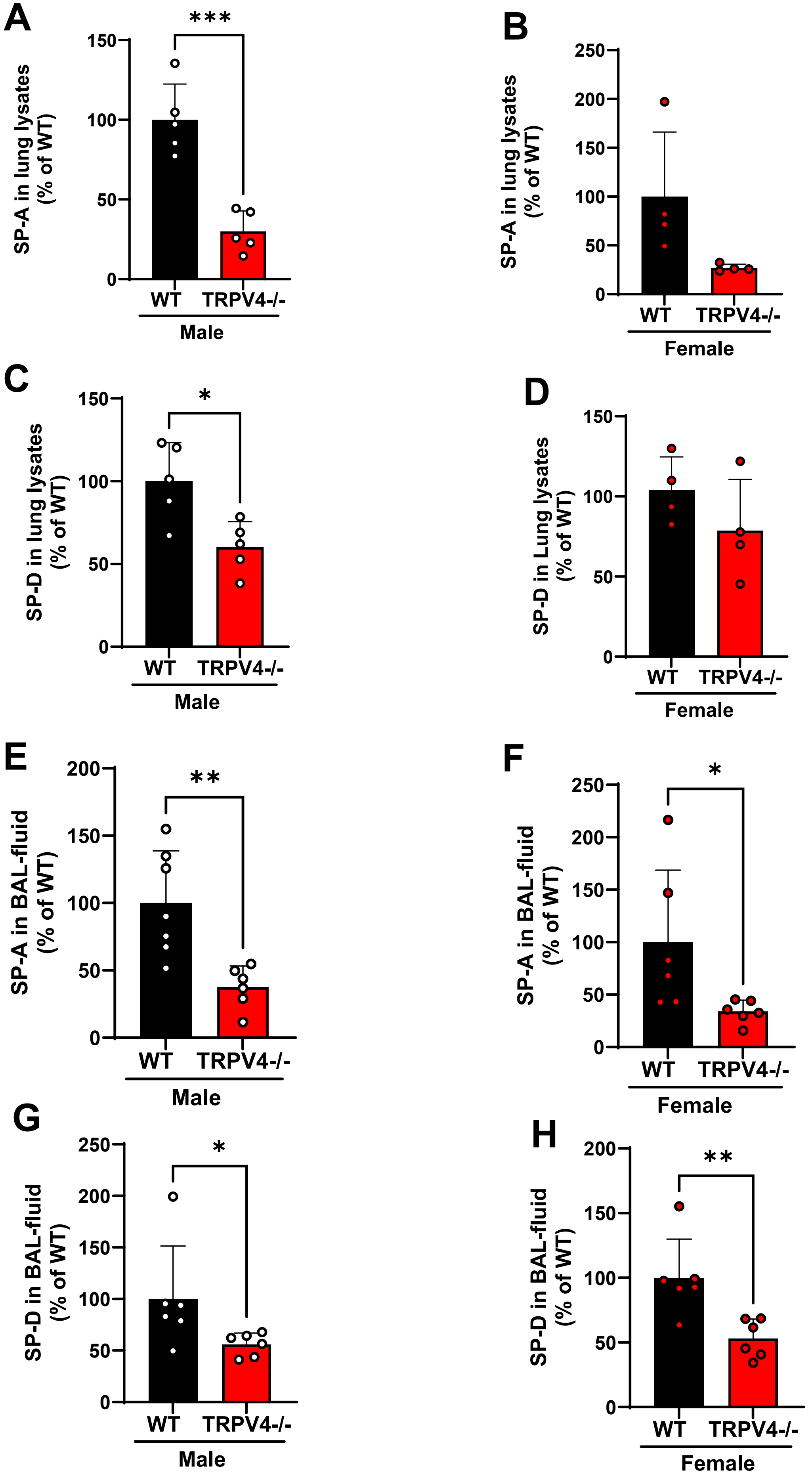

Supplemental Figure S2. Sex specific surfactant protein A (SP-A) and surfactant protein D (SP-D) levels in lung lysates and BAL fluid of WT and TRPV4-/- mice. (*A)* SP-A levels in lung lysates from male WT and TRPV4-/- mice. (*B*) SP-A levels in lung lysates from female WT and TRPV4-/- mice. (*C*) SP-D levels in lung lysates from male WT and TRPV4-/- mice. (*D*) SP-D levels in lung lysates from female WT and TRPV4-/- mice (*E*) SP-A levels in BAL fluids from male WT and TRPV4-/- mice. (*F*) SP-A levels in BAL fluids from female WT and TRPV4-/- mice. (*G*) SP-D levels in BAL fluids from male WT and TRPV4-/- mice. (*H*) SP-D levels in BAL fluids from female WT and TRPV4-/- mice. Data present means + SEM from at least 4 mice. Significance between means was analyzed using student's T-test and is indicated as ** for p<0.01 and * for p<0.05.

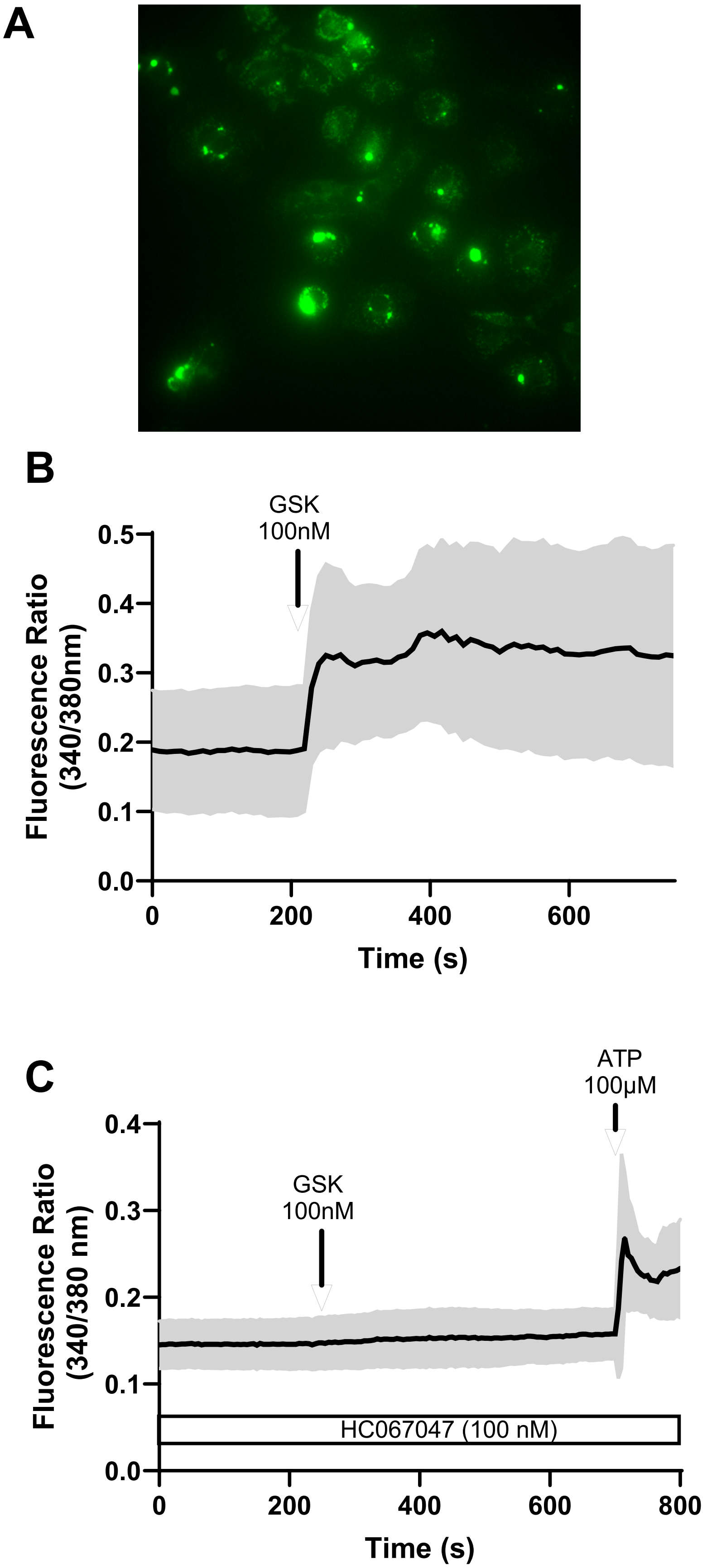

Supplemental Figure S3. Ca^{2+} imaging of murine alveolar type 2 (AT2) cells after application of a TRPV4 activator with or without preincubation with an inhibitor. (*A)* differentiated AT2 cells were identified by staining their lamellar bodies with LysoTracker DND 26 and loaded with the Ca^{2+} indicator Fura-2 AM. Fluorescence ratios were analyzed at excitation wavelengths of 340nm and 380nm. (*B*) a TRPV4 channel activator (GSK: GSK 1016790A) was applied at the indicated time point. (*C*) a TRPV4 channel inhibitor (HC067047) was applied 45 minutes before starting the analysis and a TRPV4 channel activator (GSK: GSK 1016790A) was applied at the indicted time point. ATP was added as a positive control. Data present means +/- SD for at least 7 cells in one representative analysis out of three experiments.

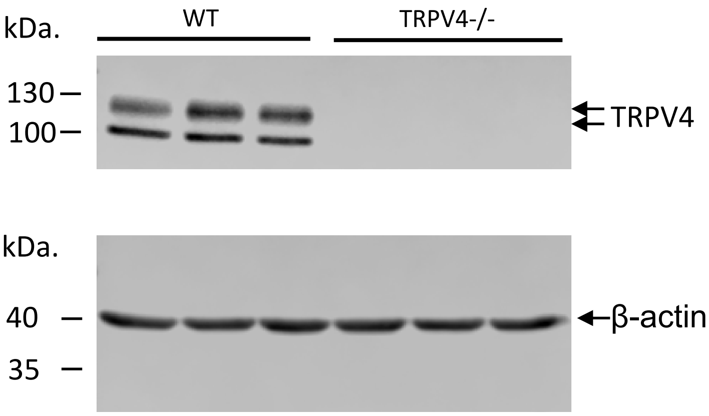

Supplemental Figure S4. Validation of TRPV4 expression in WT and TRPV4-/- mice. TRPV4 expression in cell lysates from AT2 cells 2 days after isolation. Western Blot showing three samples from each genotype representing five mice per sample (total of 15 mice each genotype). The upper part was stained with a TRPV4-specific antibody, which detects protein bands of the predicted size of TRPV4 proteins in lane 1-3. The lower part was stained with a specific β-actin antibody as loading control.

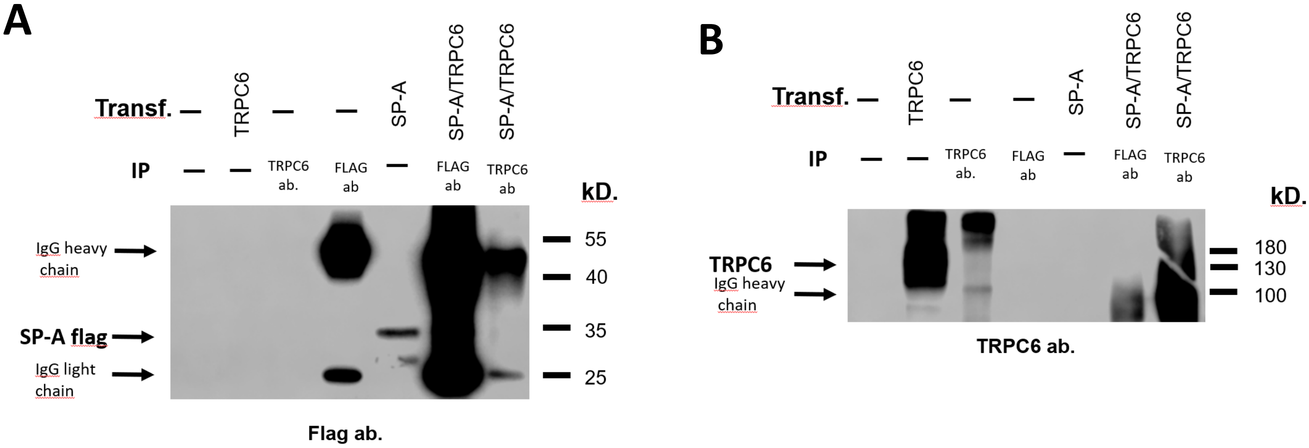

Supplemental Figure S5. Surfactant protein A (SP-A) and D (SP-D) do not physically interact with TRPC6 channels in a heterologous overexpression system. HEK293 cells were transfected with SP-A flag (SP-A), TRPC6 (TRPC6), both (SP-A/TRPV4) cDNAs or mock (-). Proteins were co-immunoprecipitated with TRPC6 or flag antibody, separated by PAA gel electrophoresis and identified in a Western Blot by (*A*) flag antibodies (Flag ab.) or (*B*) TRPC6 antibodies (TRPC6 ab.).

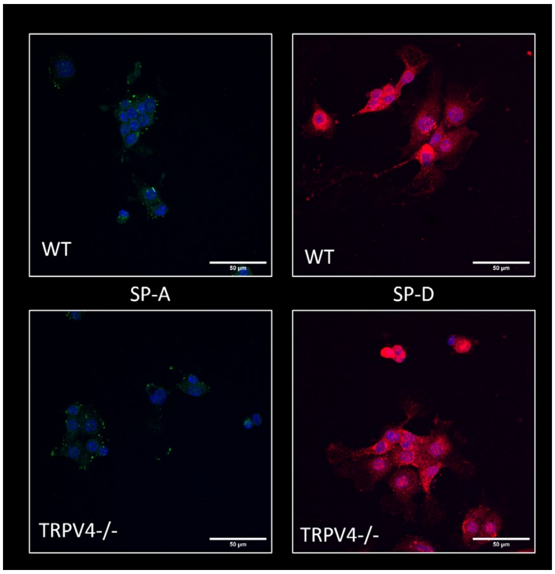

Supplemental Figure S6. Detection of SP-A and SP-D by specific antibodies in primary AT2 cells from WT and TRPV4-/- mice. Fluorescence images of AT2 cells from wild-type (WT) and TRPV4-deficient (TRPV4-/-) mice incubated with specific antibodies directed against SP-A (green fluorescence) or SP-D (red fluorescence) bound to fluorescence-coupled secondary antibodies. Nuclei were stained with DAPI (blue).

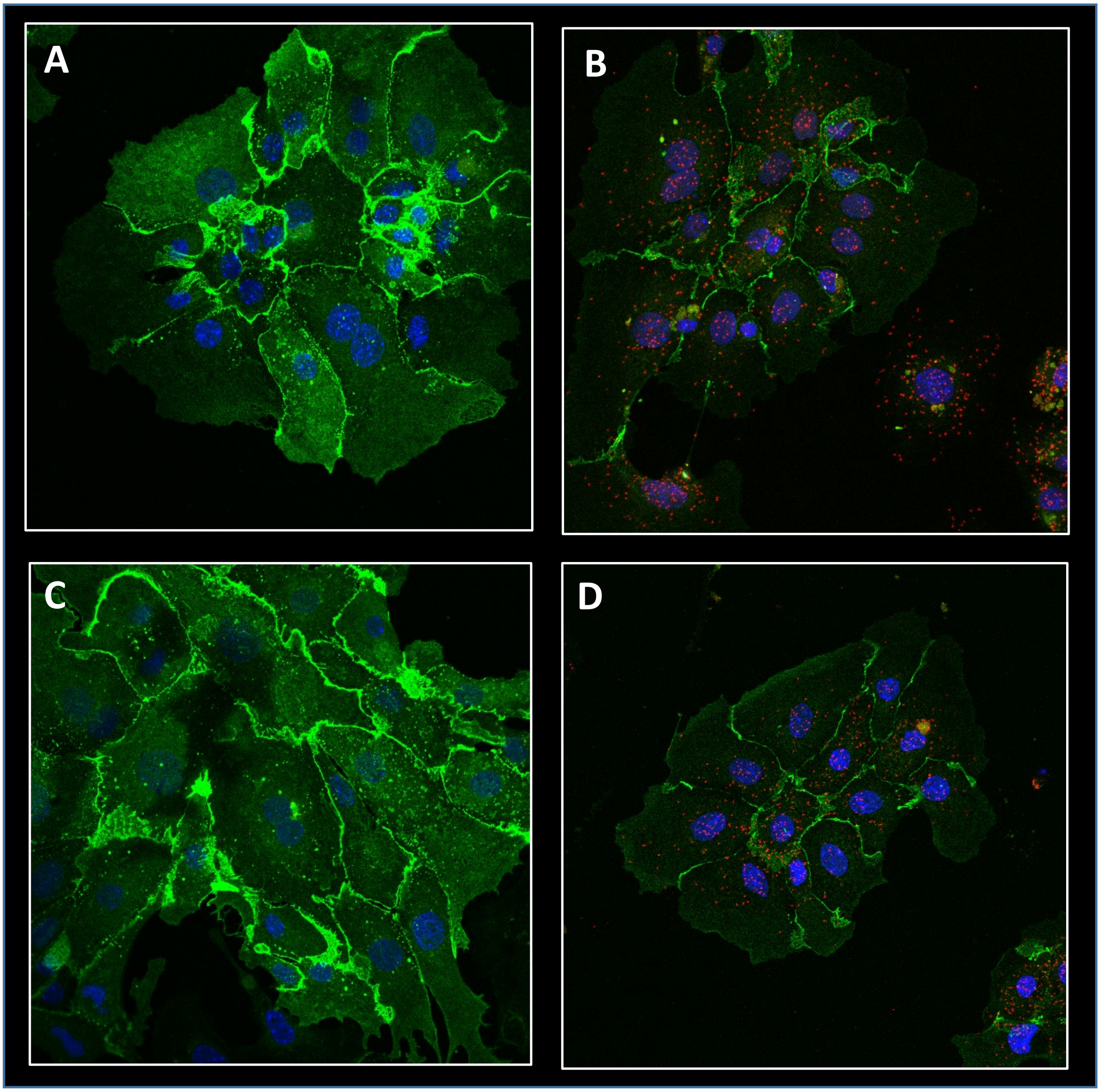

Supplemental Figure S7. Cellular localization of fluorescent signals formed by a Proximity Ligation Assay (PLA) with specific antibodies directed against TRPV4 channels and surfactant protein A (SP-A) or surfactant protein D (SP-D). (*A*) Fluorescence images of AT2 cells from wild-type mice incubated with specific antibodies directed against E-Cadherin and (*B*) after performing a PLA with antibodies directed against SP-A and TRPV4 channels. (*C*) Fluorescence images of AT2 cells from wild-type mice incubated with specific antibodies directed E-Cadherin and (*D*) after performing a PLA with antibodies directed against SP-D and TRPV4 channels. Red dots indicate interactions of TRPV4 channels with SP-A or –D. Cell nuclei were stained with DAPI in blue.

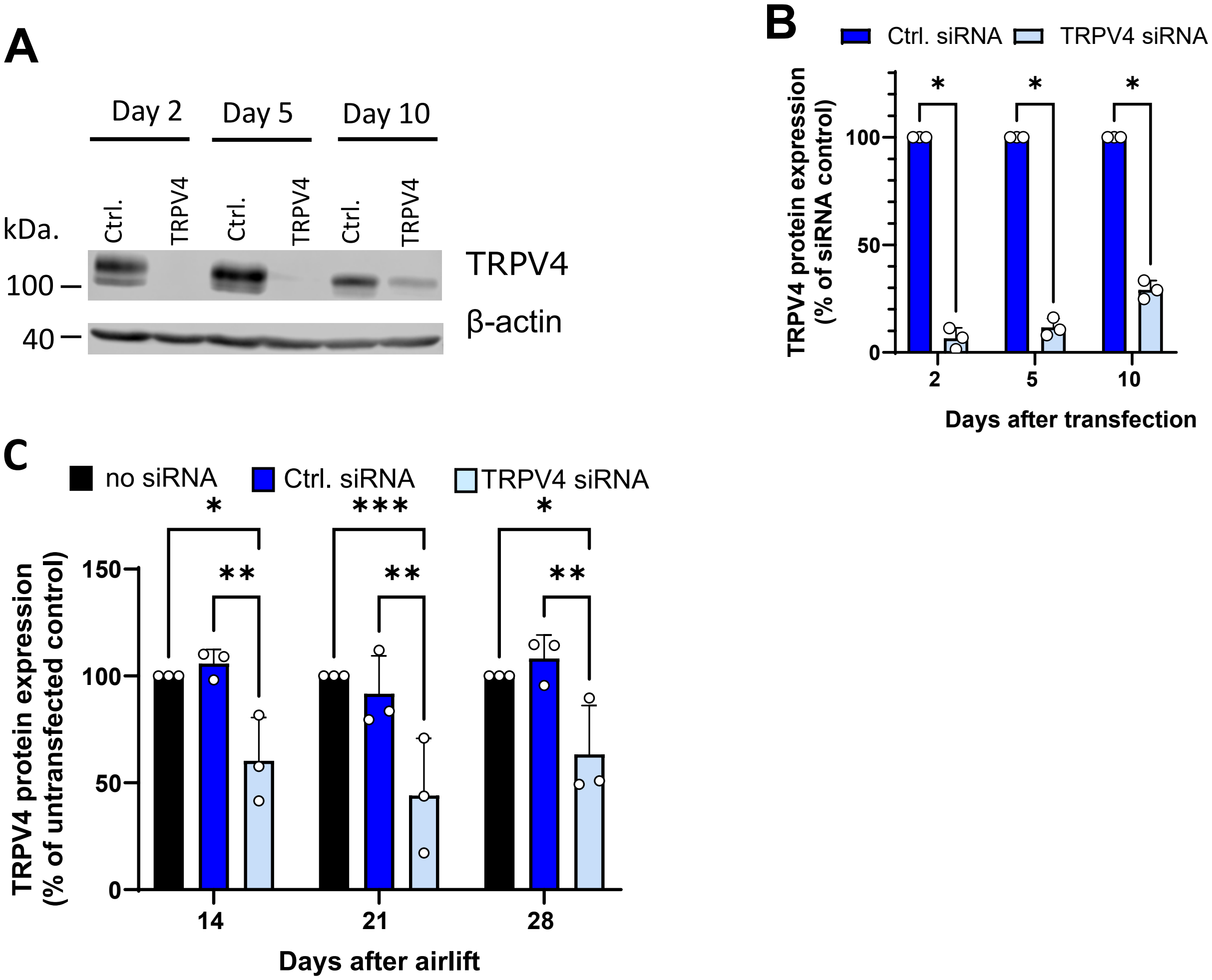

Supplemental Figure S8. Downregulation of TRPV4 channels in an Air-Liquid-Interface (ALI) model with human bronchial epithelial cells (HBEC). (*A*) Expression of TRPV4 proteins in cells transfected with control siRNAs (Ctrl.) or TRPV4 specific siRNAs (TRPV4) was quantified by Western Blotting of protein lysates of cells differentiated for the indicated time points using a TRPV4 specific antibody. β-actin served as loading control. (*B*) Quantification of TRPV4 expression after transfection of cells with control siRNAs (Ctrl. siRNA) or TRPV4 specific siRNAs (TRPV4 siRNA). Data represent means + SEM from 3 donors. Significance between means of each day was analyzed using two-tailed unpaired Student's t-test and indicated as * for p<0.05. (*C*) TRPV4 expression in cell lysates from the ALI model 14, 21 and 28 days after air-lift in untransfected (no siRNA) HBEC and cells transfected with a control siRNA (Ctrl. siRNA) or a TRPV4 specific siRNA (TRPV4 siRNA) at day 3 before air lift in % of the untransfected controls. Data present means + SEM from 3 different donors with technical duplicates. Significance between means was analyzed using two-way ANOVA and is indicated as *** for p<0.001, ** for p<0.01 and * for p<0.05.

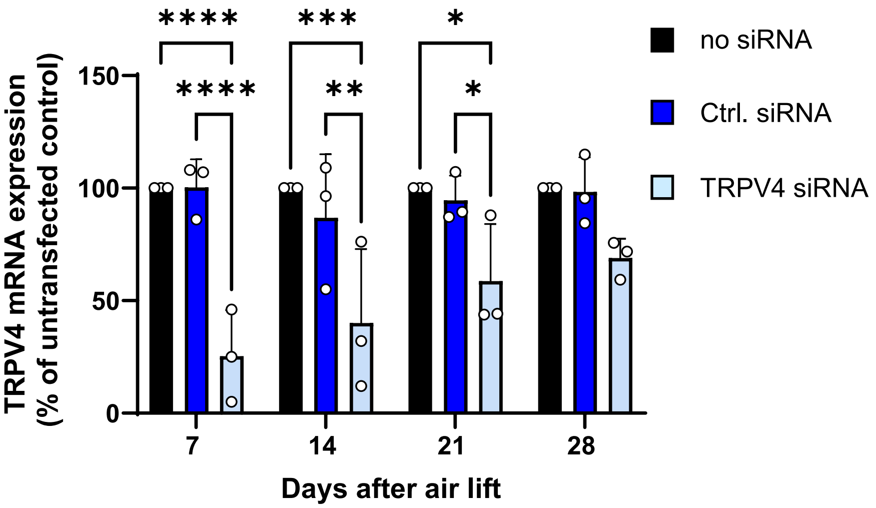

Supplemental Figure S9. Downregulation of TRPV4 mRNA in an Air-Liquid-Interface (ALI) model with human bronchial epithelial cells (HBEC). TRPV4 mRNA expression in cell lysates from the ALI model 7, 14, 21 and 28 days after air-lift in untransfected (no siRNA) HBEC and cells transfected with a control siRNA (Ctrl. siRNA) or a TRPV4 specific siRNA (TRPV4 siRNA) at day 3 before air lift in % of the untransfected controls Data present means + SEM from 3 different donors. Significance between means was analyzed using two-way ANOVA and is indicated as **** for p<0.0001, *** for p<0.001, ** for p<0.01 and * for p<0.05

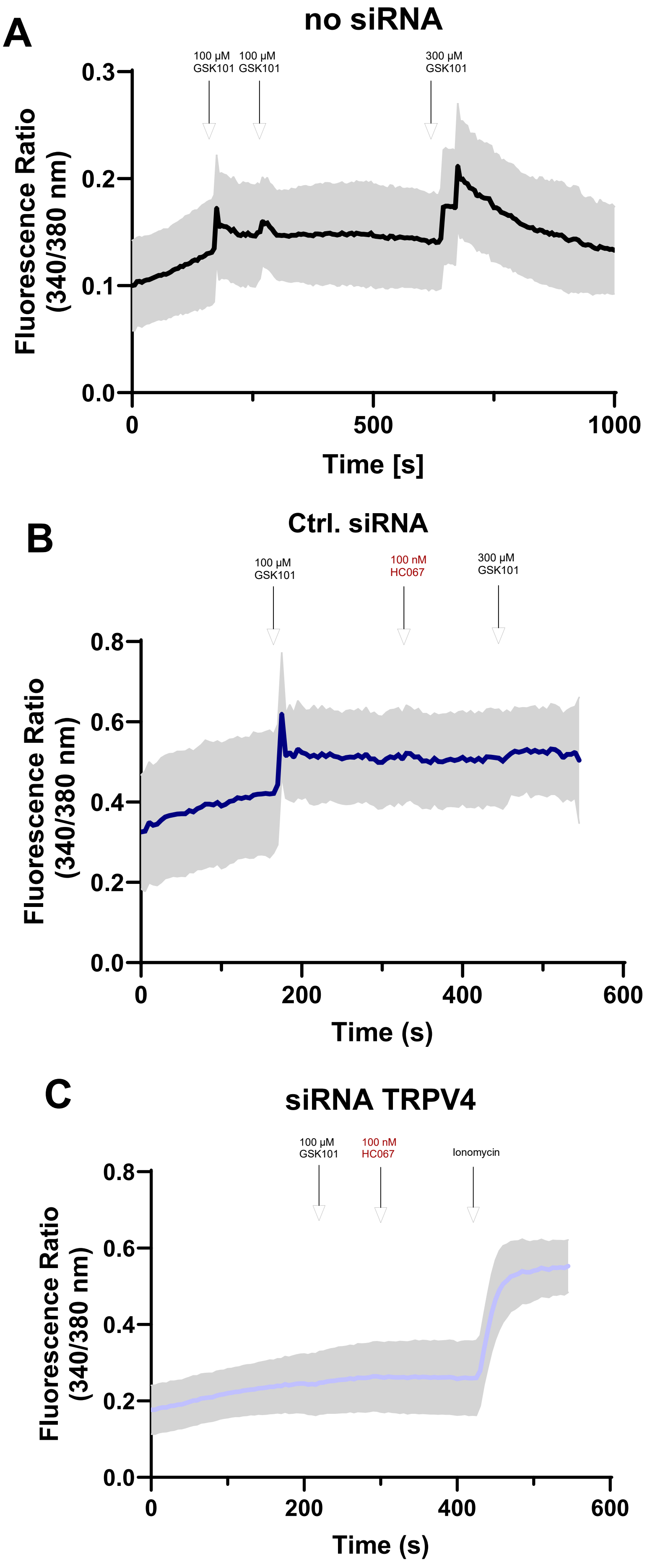

Supplemental Figure S10. Ca^{2+} imaging of human bronchial epithelial cells (HBEC) with or without downregulation of TRPV4 channels after application of a TRPV4 activator and/or inhibitor. Untransfected HBEC ((*A*) no siRNA) and cells transfected with a control siRNA ((*B*) Ctrl. siRNA) or a TRPV4 specific siRNA ((*C*) TRPV4 siRNA) were loaded with the Ca^{2+} indicator Fura-2 AM and fluorescence ratios were analyzed at excitation wave lengths of 340nm and 380nm. A TRPV4 activator (GSK 1016790A) and/or an TRPV4 inhibitor (HC067047) were applied at the indicated time points. Ionomycin (2 µM) was added as a positive control. Data present means +/- SD for at least 20 cells.

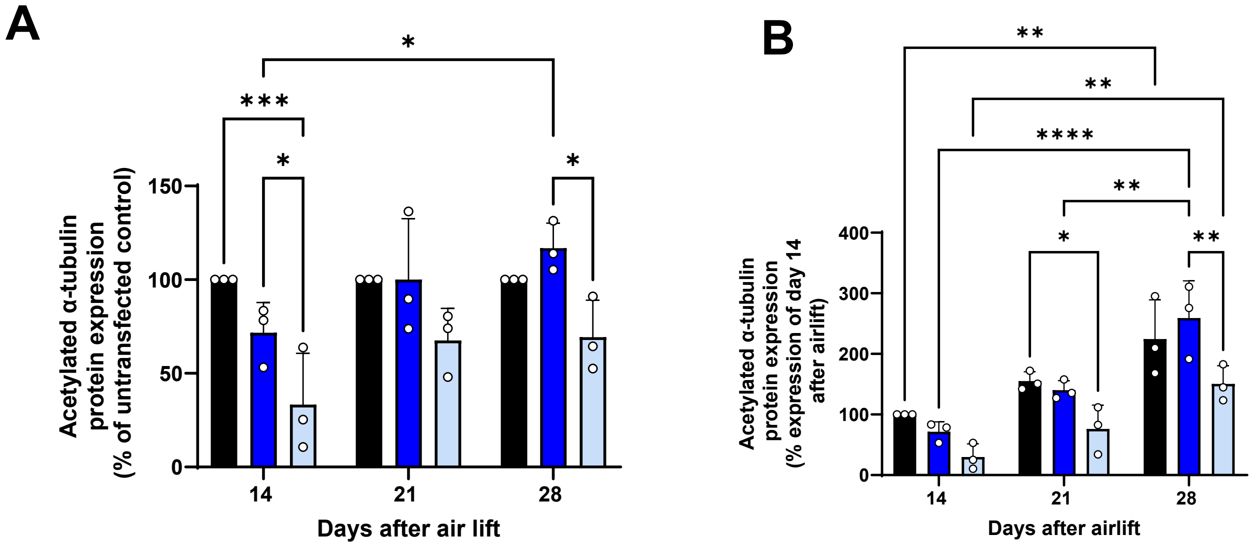

Supplemental Figure S11. Expression of acetylated α-tubulin in an Air-Liquid-Interface (ALI) model with human bronchial epithelial cells (HBEC) after down-regulation of TRPV4 channels. (A) Expression of acetylated α-tubulin in cell lysates from the ALI model 14, 21 and 28 days after air-lift in untransfected (No siRNA) HBEC and cells transfected with a control siRNA (Ctrl. siRNA) or a TRPV4 specific siRNA (TRPV4 siRNA) 3 days before air lift in % of untransfected controls. (B) Expression of acetylated α-tubulin in cell lysates from the ALI model 14, 21 and 28 days after air-lift in untransfected (No siRNA) hBEC and cells transfected with a control siRNA (Ctrl. siRNA) or a TRPV4 specific siRNA (TRPV4 siRNA) 3 days before air lift in % expression at day 14 after air-lift. Data present means + SEM from 3 different donors. Significance between means was analyzed using two-way ANOVA and is indicated as **** for $p<0.0001$ and ** for $p<0.01$.

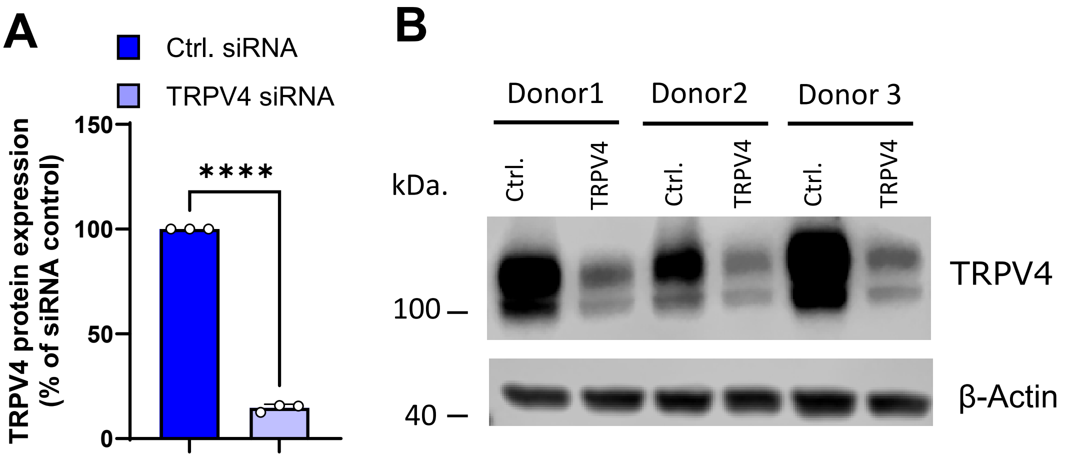

Supplemental Figure S12. Downregulation of TRPV4 channels in an Air-Liquid-Interface (ALI) model with human bronchial epithelial cells (HBEC) at day 35 after air-lift. (*A*) TRPV4 protein expression in cell lysates from the ALI model at day 35 after air-lift in HBEC transfected with a control siRNA (Ctrl. siRNA) or a TRPV4 specific siRNA (TRPV4 siRNA) 28 days after air lift in % of Ctrl. siRNA transfected cells. (*B*) Representative Western Blot of TRPV4 protein expression in cell lysates from the ALI model at day 35 after air-lift transfected with a control siRNA (Ctrl. siRNA) or a TRPV4 specific siRNA (TRPV4 siRNA) 28 days after air lift incubated with a TRPV4 specific antibody. Data present means + SEM from 3 different donors. Significance between means was analyzed using two-tailed unpaired Student's t-test and is indicated as **** for $p<0.0001$.

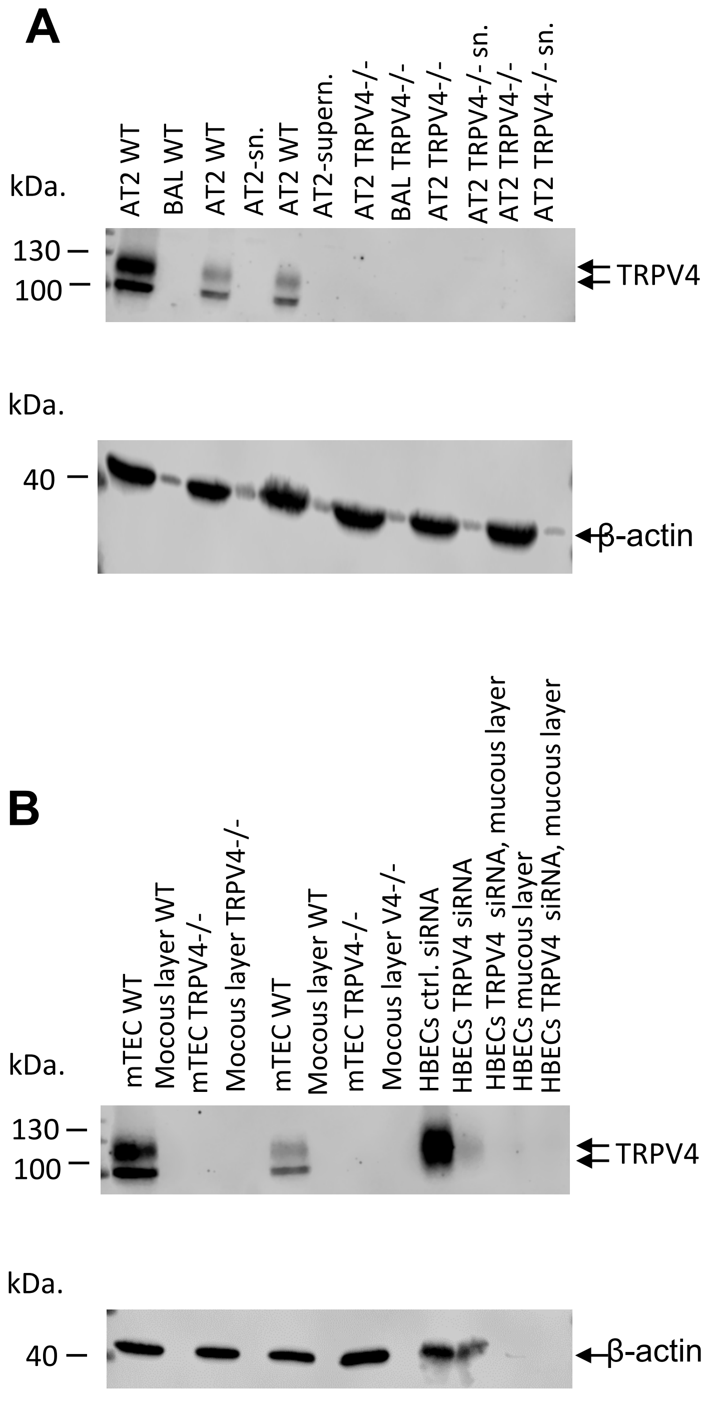

Supplemental Figure S13. Validation of TRPV4 channel expression in cell lysates, but not in the broncheoalveolar lavage (BAL) and mucous layers. (*A*) TRPV4 expression in cell lysates from wild-type (WT) and TRPV4-deficient (TRPV4-/-) AT2 cells (AT2) and broncheoalveolar lavages (BAL). (*B*) TRPV4 expression in cell lysates from wild-type (WT) and TRPV4-deficient (TRPV4-/-) murine tracheal epithelial cells (mTEC) as well as in human bronchial epithelial cells (HBECs) transfected with a control or TRPV4 specific siRNA and mucous layers. The upper part was stained with a TRPV4-specific antibody, which detects protein bands of the predicted size of TRPV4 proteins in lane 1-3. The lower part was stained with a specific β-actin antibody as loading control.
